# Identification of CD8^+^ T Cell Related Biomarkers in Ovarian Cancer

**DOI:** 10.3389/fgene.2022.860161

**Published:** 2022-05-27

**Authors:** Ling Li, Dian Chen, Xiaolin Luo, Zhengkun Wang, Hanjie Yu, Weicheng Gao, Weiqiang Zhong

**Affiliations:** ^1^ Department of Anesthesiology, Affiliated Foshan Maternity & Child Healthcare Hospital, Southern Medical University (Foshan Maternity & Child Healthcare Hospital), Foshan, China; ^2^ Division of Respiratory and Critical Care Medicine, Department of Internal Medicine, Tongji Hospital, Tongji Medical College, Huazhong University of Science and Technology, Wuhan, China; ^3^ Department of Anesthesiology, Sun Yat-sen University Cancer Center, State Key Laboratory of Oncology in Southern China, Collaborative Innovation for Cancer Medicine, Guangzhou, China; ^4^ 3D Medicines, Inc., Shanghai, China; ^5^ Department of Urology, The First Affiliated Hospital of Guangdong Pharmaceutical University, Guangzhou, China

**Keywords:** ovarian cancer (OC), CD8^+^ T cell, immunotherapy, CXCL13, prognosis

## Abstract

**Background:** Immunotherapy is a promising strategy for ovarian cancer (OC), and this study aims to identify biomarkers related to CD8^+^ T cell infiltration to further discover the potential therapeutic target.

**Methods:** Three datasets with OC transcriptomic data were downloaded from The Cancer Genome Atlas (TCGA) and Gene Expression Omnibus (GEO) databases. Two immunotherapy treated cohorts were obtained from the Single Cell Portal and Mariathasan’s study. The infiltration fraction of immune cells was quantified using three different algorithms, Cell-type Identification by Estimating Relative Subsets of RNA Transcripts (CIBERSORT), and microenvironment cell populations counter (MCPcounter), and single-sample GSEA (ssGSEA). Weighted gene co-expression network analysis (WGCNA) was applied to identify the co-expression modules and related genes. The nonnegative matrix factorization (NMF) method was proposed for sample classification. The mutation analysis was conducted using the “maftools” R package. Key molecular markers with implications for prognosis were screened by univariate COX regression analysis and K-M survival analysis, which were further determined by the receiver operating characteristic (ROC) curve.

**Results:** A total of 313 candidate CD8^+^ T cell-related genes were identified by taking the intersection from the TCGA-OV and GSE140082 cohorts. The NMF clustering analysis suggested that patients in the TCGA-OV cohort were divided into two clusters and the Cluster 1 group showed a worse prognosis. In contrast, Cluster 2 had higher amounts of immune cell infiltration, elevated ssGSEA scores in immunotherapy, and a higher mutation burden. CSMD3, MACF1, PDE4DIP, and OBSCN were more frequently mutated in Cluster 1, while SYNE2 was more frequently mutated in Cluster 2. CD38 and CXCL13 were identified by univariate COX regression analysis and K-M survival analysis in the TCGA-OV cohort, which were further externally validated in GSE140082 and GSE32062. Of note, patients with lower CXCL13 expression showed a worse prognosis and the CR/PR group had a higher expression of CXCL13 in two immunotherapy treated cohorts.

**Conclusion:** OC patients with different CD8^+^ T cell infiltration had distinct clinical prognoses. CXCL13 might be a potential therapeutic target for the treatment of OC.

## Introduction

Among the most commonly diagnosed gynecological malignancies worldwide, ovarian cancer (OC) remains the leading cause of cancer death with the highest mortality (Moss et al., 2018; Yang et al., 2018; Li et al., 2019). The estimated new cases of OC in 2021 are expected to be 21,410 with 13,770 estimated deaths in the United States, reported in SEER Cancer Stat Facts (https://seer.cancer.gov/statfacts/html/ovary.html) (Institute, 2021). Over 70% of OC patients are diagnosed at advanced stages with a staggering low five-year survival rate of approximately 49.1% despite maximal treatment improvements (Institute, 2021). Primary cytoreductive surgery combined with platinum-based chemotherapy is now recommended as the optimal and standard treatment available to patients with advanced OC, however, the treatment landscape has revolutionized to lengthen survival and improve quality of life within the scope of poly ADP-ribose polymerase (PARP) inhibitors (Moore et al., 2018; Coleman et al., 2019; González-Martín et al., 2019), folate receptor antibody-drug conjugates (O'Malley et al., 2020), chimeric antigen receptor therapy (Coon et al., 2020), and immunotherapy (Hamanishi et al., 2015; Liu et al., 2019; Zamarin et al., 2020). Of note, immunotherapy has emerged as a standard pillar of modern cancer treatment, nevertheless, a proportion of patients with OC respond poorly, highlighting the need of exploring the immune-related molecular markers.

Multiple immunotherapeutic strategies have been exploited to treat OC, including acting directly on the OC cells, targeting the tumor microenvironment (TME), and enhancing the host immune system (Lheureux et al., 2019a). Numerous studies have revealed the role of TME in promoting tumor development, metastasis, and altering the response to immunotherapy. In addition, the major obstacle to clinical efficacy for successful immunotherapy is primarily limited by an immunosuppressive TME (Gordon-Alonso et al., 2017). The intricate interplay between tumor-infiltrating lymphocytes (TILs) and cancer’s genomic changes is strongly associated with clinical outcomes in OC patients, among which CD8^+^ cytotoxic T lymphocytes (CTLs) are the main players in mediating cytotoxic killing of cancer cells in most immunotherapy settings (Lheureux et al., 2019b; Desbois et al., 2020).

Previous studies have demonstrated the prognostic significance of tumor-infiltrating CD8^+^ T cells in OC and the presence of CD8^+^ T cells is associated with a good clinical outcome (Zhang et al., 2003; Hwang et al., 2012; Tiper et al., 2016; Goode et al., 2017). The combination of checkpoint inhibitors with PARP inhibitors has been involved in a few studies, where PARP inhibitors were proved to activate and synergize PD-1 and CTLA-4 blockades in a mouse model (Wang et al., 2019). Meanwhile, in phase II clinical trial, the combination of the PARP inhibitor niraparib with an anti-PD-1 antibody pembrolizumab confirmed a promising antitumor activity for OC patients with limited treatment options (Konstantinopoulos et al., 2019). Despite CTLA-4 and PD-1 targeting checkpoint inhibitors, PVRL2 is highly expressed in OC and the antagonism of its receptor significantly increased CD8^+^ T cell cytokine production and cytotoxic activity (Whelan et al., 2019). Therefore, the induction of CD8^+^ T cell infiltration and the identification of related biomarkers are in established need for immunotherapies to improve survival outcomes for the broader population of OC patients.

Weighted gene co-expression network analysis (WGCNA) is a widely used method in screening candidate biomarkers and exploring the relationships between gene sets and external biological clinical traits (Langfelder and Horvath, 2008). Multivarious research has utilized this algorithm to identify hub genes involved in the pathogenesis of OC (Zeleznik et al., 2020; Chang et al., 2021; Quan et al., 2021). Another notable bioinformatics tool based on support vector regression modeling, Cell-type Identification by Estimating Relative Subsets of RNA Transcripts (CIBERSORT), was developed to deconvolute cell types and dissect the cellular components at the transcription level (Newman et al., 2015). This algorithm has been extensively used to investigate the immune infiltration fraction and the heterogeneity of the immune microenvironment in OC tissues (An and Yang, 2020; Cong et al., 2020; Gao et al., 2020).

In our study, the CIBERSORT algorithm was first used to calculate the immune cell compositions, followed by the WGCNA analysis to evaluate the function of CD8^+^ T cells in the TME and identify related potential biomarkers. Consensus clustering analysis was then performed for OC patients based on CD8^+^ T cell-related genes and two distinct subtypes with different survival outcomes were identified. CD38 and CXCL13 were further verified to carry prognostic significance. Noteworthy, CXCL13 showed a good performance in two immunotherapy treated cohorts, illustrating the potential value of CXCL13 as a therapeutic target in OC. Our seminal discovery first employed WGCNA to identify CD8^+^ T cell-related biomarkers in OC and provided new clinical treatment guidelines for clinicians.

## Methods

### Data Acquisition and Processing

Transcriptome and clinical data for OC patients were retrieved from the TCGA data portal using the “TCGAbiolinks” R package (Colaprico et al., 2016). A total of 353 OC samples were involved in the study after the initial quality control. The FPKM expression units were converted into TPM units for further analyses. The survival data of the TCGA-OV dataset was downloaded from the UCSC Xena platform (https://xena.ucsc.edu/). The raw gene expression profiles of GSE140082 and GSE32062 were acquired from the Gene Expression Omnibus (GEO) database by using the R package “GEOquery” (Davis and Meltzer, 2007). 380 samples were involved in the GSE140082 profile and 260 samples were involved in the GSE32062 after matching clinical data, respectively. GSE115978 and IMvigor210 were immunotherapy treated cohorts. The single-cell data set GSE115978 was available through the Single Cell Portal (https://singlecell.broadinstitute.org/single_cell). The complete annotated expression and clinical data of the IMvigor210 cohort were obtained from the study of Sanjeev Mariathasan under Creative Commons 3.0 License (Mariathasan et al., 2018). A total of 348 patients after immunotherapy treatment was involved after matching clinical data. More information on these datasets we utilized could be found in the supplementary materials.

### Construction of Weighted Gene Co-Expression Network

The expression profiles of the TCGA-OV and GSE140082 cohorts were integrated with the relative proportions of CD8^+^ T cell populations to construct a weighted gene co-expression network by using the R package “WGCNA” (Langfelder and Horvath, 2008). The similarity matrix was first characterized given Pearson’s correlation value and then converted into an adjacency matrix, as selected by the weighting coefficient, ß. Subsequently, the adjacency matrix was transformed into a topological overlap matrix, followed by utilizing the dynamic tree cut method to recognize various modules with a module least size cutoff of 50.

### Construction of Module Trait Relationships

The correlations between modules and clinical information (the infiltration level of CD8^+^ T cells, OS time, and OS statue) were investigated to determine the significance of modules by Pearson correlation’s analysis. A module was considered to have a significant correlation with CD8^+^ T cells while p-value was <0.05. The module with the highest correlation coefficient was then defined as the hub module.

### Non-Negative Matrix Factorization Classification and Prognostic Analysis of Ovarian Cancer Patients

The TCGA-OV patients were separated into different subtypes by performing non-negative matrix factorization (NMF) with the “brunet” standard and 50 iterations (Brunet et al., 2004; Gao and Church, 2005). The number of clusters, k, was set as 2 to 10, and the average contour width of the common member matrix was determined by the “NMF” package in R software. The minimum member of each subtype was set to 10. The optimal number of clusters was determined according to cophenetic, dispersion, and silhouette coefficients. The overall survival and progress-free survival of OC patients in the different clusters were determined by Kaplan-Meier (K-M) survival analysis. P values were calculated by the Log-rank test.

### Analysis of Immune Infiltrating Cells in Tumor Tissues

Quantification of immune infiltration was performed using three different methods. The CIBERSORT algorithm was used to calculate the proportion of infiltrating immune cell subsets (Newman et al., 2015). The ratio of the immune-stromal component in the TME under different clusters of OC patients was estimated by the Estimation of STromal and Immune cells in MAlignant Tumor tissues using the Expression data (ESTIMATE) algorithm (Computing, 2018), while the populations of eight immune cells and two stromal cells were calculated using the microenvironment cell populations counter (MCPcounter) method (Becht et al., 2016). Single-sample GSEA (ssGSEA) method was used to evaluate the functions of 12 immunotherapy-related gene sets in each sample of the TCGA-OV cohort (Hänzelmann et al., 2013). Wilcox test was used to analyze the statistical significance of differences between two groups, and the Kruskal test was used to compare differences among multiple groups. Spearman’s correlation test was used to perform the correlation analysis.

### Enrichment Analyses of Ovarian Cancer Subtypes

Gene ontology annotation, KEGG pathway, and GSEA analyses were conducted using the Clusterprofiler R package (Yu et al., 2012). For GO and KEGG, the enrichment analyses were performed on DEGs and only terms with P-value < 0.05 were considered significantly enriched. For GSEA, c2.cp.kegg.v7.4.symbols.gmt file was downloaded from the Molecular Signature Database as the target set to identify enriched KEGG pathways in distinct OC clusters. Only |NES| > 1 and FDR <0.05 were considered statistically significant.

### Analysis of Somatic Mutations

Somatic mutations of the TCGA-OV patient cohort were retrieved from the online website cBioPortal (http://www.cbioportal.org/). For different mutational types, FRAME_SHIFT_DEL, FRAME_SHIFT_INS, In_FRAME_DEL, In_FRAME_INS, MUSSENSE, NOTHINE, NONSTOP, Splice_SITE, and TRANSPOT_START_SITE was classified as non-synonymous variants, while silent mutations and other mutation types were classified as synonymous variants, including Intron, 3′ UTR, 5′ UTR, 3′ Flank, 5’ Flank, IGR, RNA, and Splice_Region. Subsequently, the somatic mutation data were visualized by the “maftools” R package (Mayakonda et al., 2018), which was also used to evaluate mutated genes and calculate tumor mutational burden (TMB). The proportions of mutations were compared using a one-sided z test and a two-sided chi-squared test.

### Clinical Significance Analysis of CD8^+^ T Cell Related Genes in Ovarian Cancer

Univariate COX proportional hazards regression analysis was executed using the R package “survival”. Survival analysis and survival curves were conducted and plotted using R packages “survival” and “survminer” (Therneau and Grambsch, 2000). For certain gene expression, patients with values above and below the median were classified as “high expression” and “low expression” groups, respectively. Log-rank test was used to calculate the significance of differences in overall survival. Receiver operating characteristic (ROC) curves were plotted by combining normal tissue RNA sequencing data from the Genotype-Tissue Expression (GTEx) database with the data from the TCGA-OV cohort using R package pROC (Robin et al., 2011).

### Statistical Analyses

Detailed statistical analyses of bioinformatics were described above. The Association of the mutation rate between two clusters was evaluated by Fisher’s exact test. One-sided z test and chi-square test were used to compare the continuous and categorical variables between two clusters. For survival analysis, the Kaplan-Meier method and Log-rank test were employed. Spearman’s correlation test was implemented in correlation analysis. P values <0.05 were considered significant (*: *p* < 0.05; **: *p* < 0.01). Most analyses above were done by packages of R software (version 3.6.3), aside from a little piece of one that was performed joining with R version 4.1.0.

## Results

### Construction of Gene Co-Expression Network and Identification of Hub Modules

**FIGURE 1:**
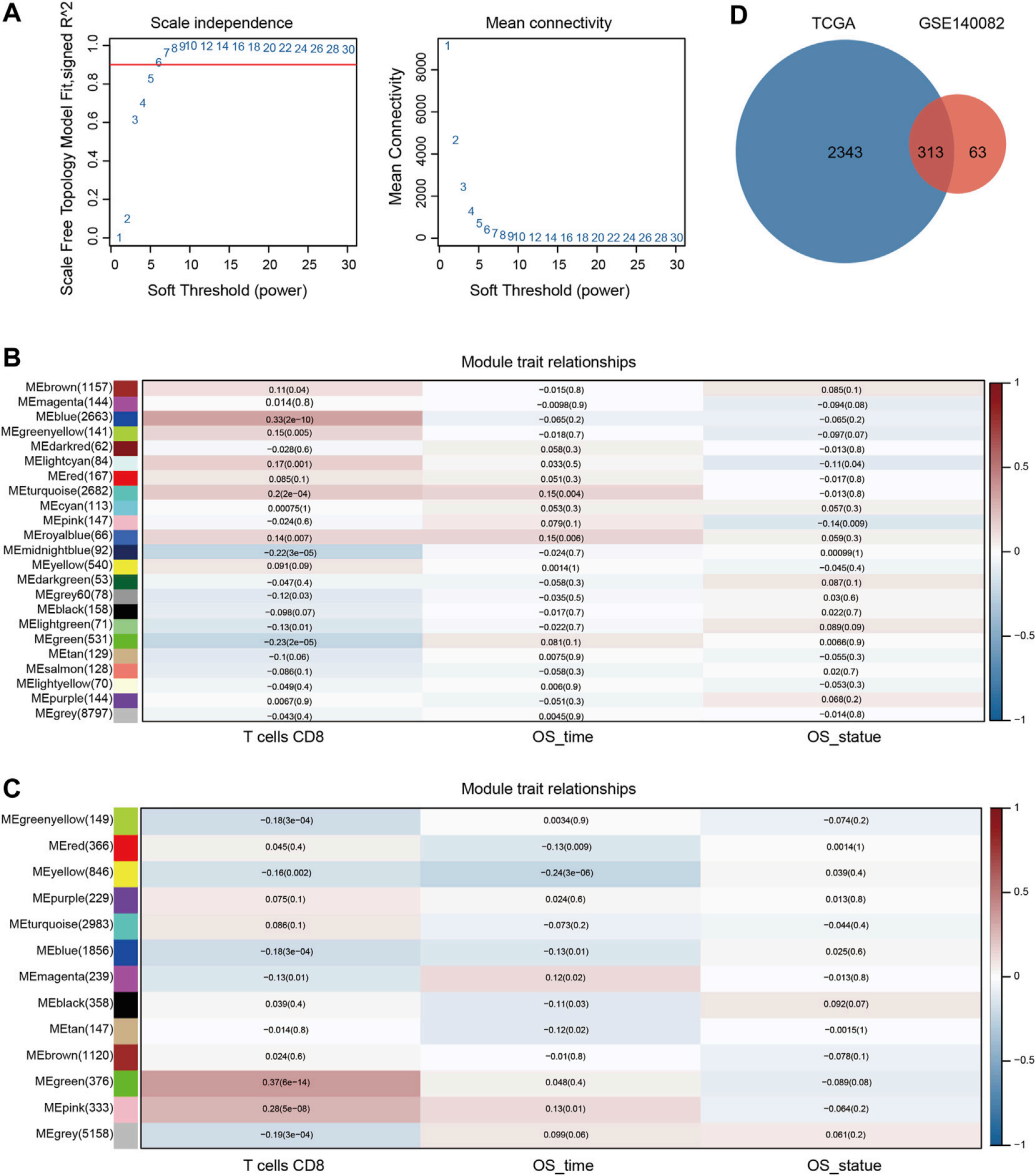
Identification of key modules correlated with OC *via* WGCNA. **(A)** Analysis of the scale-free fit index and the average connectivity of various soft threshold power. Heatmap of the association between module eigengenes and status (CD8^+^ T cell infiltration, OS time, and OS statue) in the TCGA-OV **(B)** and GSE140082 **(C)** cohort. The corresponding correlation coefficient and P value were presented in each cell. **(D)** The intersection of CD8^+^ T cell related genes from the two cohorts.

To quantify the relative proportions of CD8^+^ T cell populations in human OC samples, the CIBERSORT algorithm was applied to analyze the gene expression profiles of TCGA-OV and GSE140082 cohorts to infer immune infiltration. As shown in Supplementary Figure S1, the relative abundance of 22 distinct immune cell types was estimated in two cohorts. To address the complex regulatory processes involved in CD8^+^ T cell responses, we leveraged the WGCNA approach to reveal correlated modules in two cohorts and uncover associated clinical traits. To guarantee a scale-free network, the power of ß = 6 and 3 were selected as the soft-threshold value in TCGA-OV and GSE140082 cohorts, respectively (Figure 1A, Supplementary Figures S2, S3). In the TCGA-OV cohort, 23 regulatory gene modules were identified and the blue module showed the highest positive correlation coefficient with CD8^+^ T cell (Figure 1B). Highly correlated genes within the same module may represent similar expression patterns, biological processes, or mechanisms of regulation, therefore, 2663 genes in the blue module were selected for further screening and analysis in the TCGA-OV cohort. Similarly, in the GSE140082 cohort, 13 co-expression modules were obtained and 376 genes in the green module showed the highest correlation with CD8^+^ T cells (Figure 1C). After the intersection of CD8^+^ T cell-related genes from the two cohorts, a total of 313 candidate genes were obtained, which were further considered to be highly correlated with CD8^+^ T cell function (Figure 1D).

### Identification of CD8^+^ T Cell-Based Molecular Subtypes of Ovarian Cancer With Prognostic Significance

**FIGURE 2:**
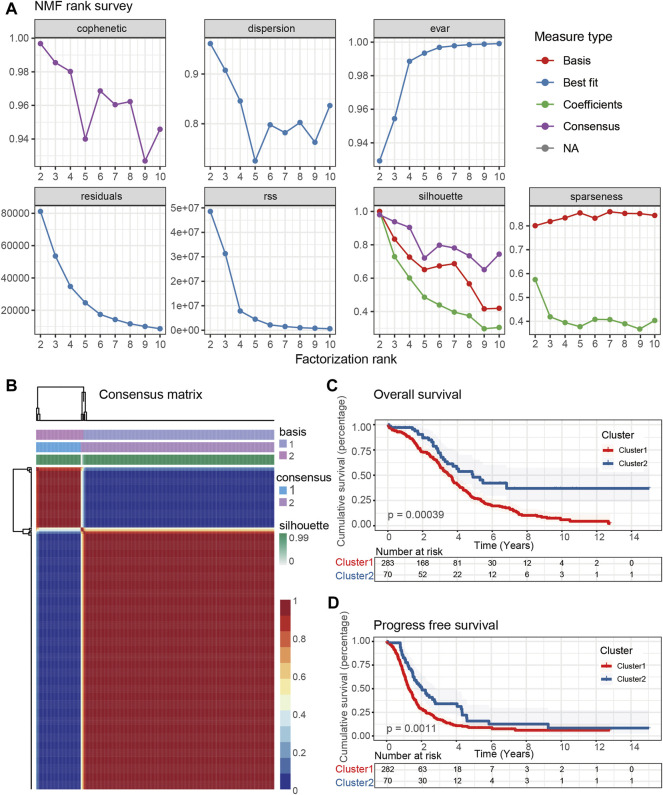
Identification of the molecular subtypes of OC patients using NMF. **(A)** The cophenetic, dispersion, evar, residuals, rss, silhouette, and sparseness distributions when rank k was set as 2 to 10. **(B)** Consensus map of NMF clustering for k = 2, which was the optimal cluster number in the TCGA-OV cohort. Kaplan-Meier (K-M) survival analyses were used to determine the OS **(C)** and PFS **(D)** prognosis of OC patients in two sub-consensuses classifications.

The mRNA levels of above 313 CD8^+^ T cell-related genes from the expression matrix of 353 OC patients in the TCGA-OV cohort were extracted to perform NMF, which was used to investigate a novel CD8^+^ T cell-based molecular classification of OC. The optimal number of clusters was set to k = 2 according to the cophenetic, dispersion, rss, and silhouette analyses (Figure 2A). Thereafter, two distinctive OC clusters were determined, including 283 patients in Cluster 1 and 70 patients in Cluster 2. The consensus map showed a clear and sharp boundary when k = 2, indicating a high correlation of OC patients in each sub-consensus (Figure 2B). Consensus maps were presented at K values of 3-10 (Supplementary Figure S4). We further used PCA and t-SNE to perform dimensionality reduction and the result revealed two main clusters, demonstrating the robustness of these clusters (Supplementary Figure S5). In addition, the K-M survival curve showed that overall survival (OS) and progress free survival (PFS) rates of Cluster 1 and Cluster 2 were significantly different (*p* < 0.05), and the Cluster 1 group showed a worse prognosis than the Cluster 2 group (Figures 2C,D).

### Analysis of the Immune Microenvironment of Subtypes of Ovarian Cancer

**FIGURE 3:**
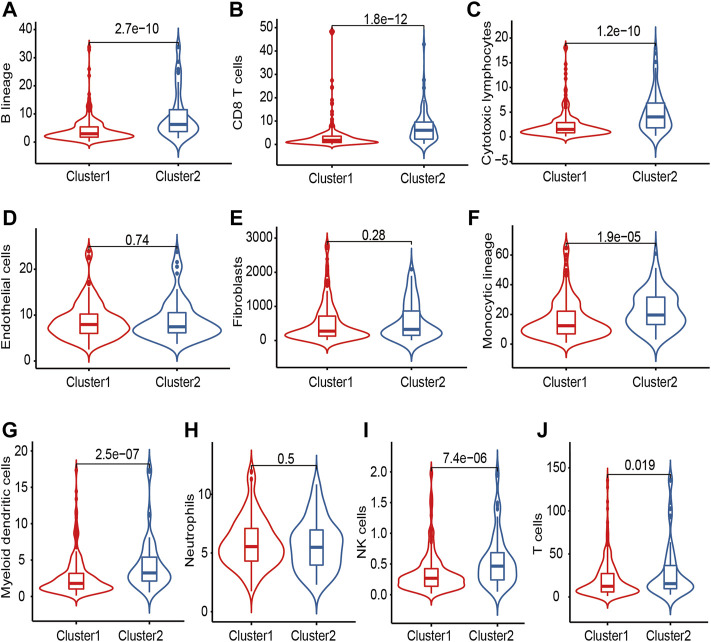
The immune cell distribution in OC patients of two clusters. **(A)** B cells; **(B)** CD8^+^ T cell; **(C)** Cytotoxic lymphocytes; **(D)** Endothelial cells; **(E)** Fibroblasts; **(F)** Monocytic cells; **(G)** Myeloid dendritic cells; **(H)** Neutrophils; **(I)** Natural killer cells; **(J)** T cells.

The intercellular interactions in the TME could induce the changes in phenotype and biological features of many types of immune cells, especially antitumor T cell responses. To clarify the difference in microenvironment composition in the two clusters, we next applied the ESTIMATE algorithm to calculate the immune score, stromal score, ESTIMATE score, and tumor purity in the TCGA-OV cohort (Supplementary Figure S6). Cluster 2 showed elevated immune score (*p* < 0.001), stromal score (*p* = 0.012), and ESTIMATE score (*p* < 0.001), but lower tumor purity (*p* < 0.001), indicating a larger amount of immune and stromal components with more infiltrating normal cells. We next used the MCPcounter tool to quantify different immune cell populations and compare the immune scores of the two clusters in the TCGA-OV cohort. As was shown in Figure 3, several types of immune cells, including B lineage (*p* < 0.001), CD8^+^ T cell (*p* < 0.001), cytotoxic lymphocytes (*p* < 0.001), monocytic lineage (*p* < 0.001), myeloid dendritic cells (*p* < 0.001), natural killer (NK) cells (*p* < 0.001), and T cells (*p* = 0.019), showed higher abundance in Cluster 2. Of note, elevated tumor-infiltrating NK and CD8^+^ T killer lymphocytes indicated increasing anti-tumor functions of Cluster 2, which was consistent with a better prognosis (Figures 2C,D).

**FIGURE 4:**
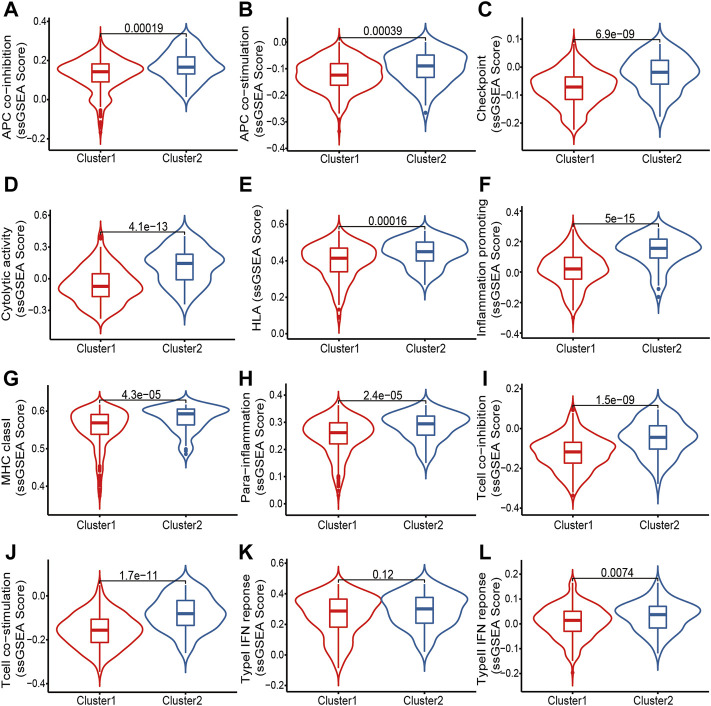
Comparisons of ssGSEA scores of 12 immunotherapy-related gene sets in two clusters of OC patients. **(A)** APC co-inhibition; **(B)** APC co-stimulation; **(C)** Checkpoint; **(D)** Cytolytic activity; **(E)** HLA; **(F)** Inflammation promoting; **(G)** MHC class I; **(H)** Para-inflammation; **(I)** T cell co-inhibition; **(J)** T cell co-stimulation; **(K)** Type I IFN response; **(L)** Type II IFN response.

The chemokine system and other immunomodulators are required in recruiting T cell infiltration into the TME. We compiled information on 148 immunomodulators from Charoentong’s study, including chemokine, MHC, receptor, immune-stimulator, and immune-inhibitor (Charoentong et al., 2017). The comprehensive analysis of immune landscapes between clusters revealed more immune-activated Cluster 2 with significantly elevated chemokines with receptors, and immune activators (Supplementary Figure S7). We thus wondered if Cluster 2 would be more sensitive to certain immunotherapeutic strategies. We further collected 12 immunotherapy-related gene sets from previously published literature and the Molecular Signatures Database (MSigDB), including APC co-inhibition, APC co-stimulation, Checkpoint, Cytolytic activity, HLA, Inflammation-promoting, MHC class I, Para-inflammation, T cell co-inhibition, T cell co-stimulation, Type I IFN Response, and Type II IFN Response. ssGSEA was then employed to score each sample to evaluate the activity of these functions (Figure 4). Not surprisingly, Cluster 2 showed significantly elevated ssGSEA scores of these 12 gene sets, further indicating the potential effect of immunotherapies.

### Functional Enrichment Analysis

We conducted GO term and KEGG pathway enrichment analyses to explore the biological functions and pathways related to different OC clusters. As shown in Supplementary Figures S8A,B, differentially expressed genes (DEGs) between CD8^+^ T cell-based molecular subtypes were mainly enriched in T cell activation, external side of the plasma membrane, and chemokine receptor binding in the biological process, cellular component, and molecular function categories, respectively. KEGG pathway analysis revealed enrichment in cytokine-cytokine receptor interaction, chemokine signaling pathway, natural killer cell-mediated cytotoxicity, and PD-L1 expression and PD-1 checkpoint pathway in cancer. Further subtype characterization was performed with GSEA analysis (Supplementary Table S1; Supplementary Figures S8C,D). DEGs in Cluster 1 were mainly enriched in metabolic pathways, pathways in cancer, and pathways of neurodegeneration-multiple diseases, while DEGs in Cluster 2 were mainly enriched in natural killer cell-mediated cytotoxicity, T cell receptor signaling pathway, and Th1 and Th2 cell differentiation. These results demonstrated distinct tumor microenvironmental effects between CD8^+^ T cell-based molecular subtypes, which was consistent with the previous conclusion.

### Mutation Analysis of Subtypes of Ovarian Cancer

**FIGURE 5:**
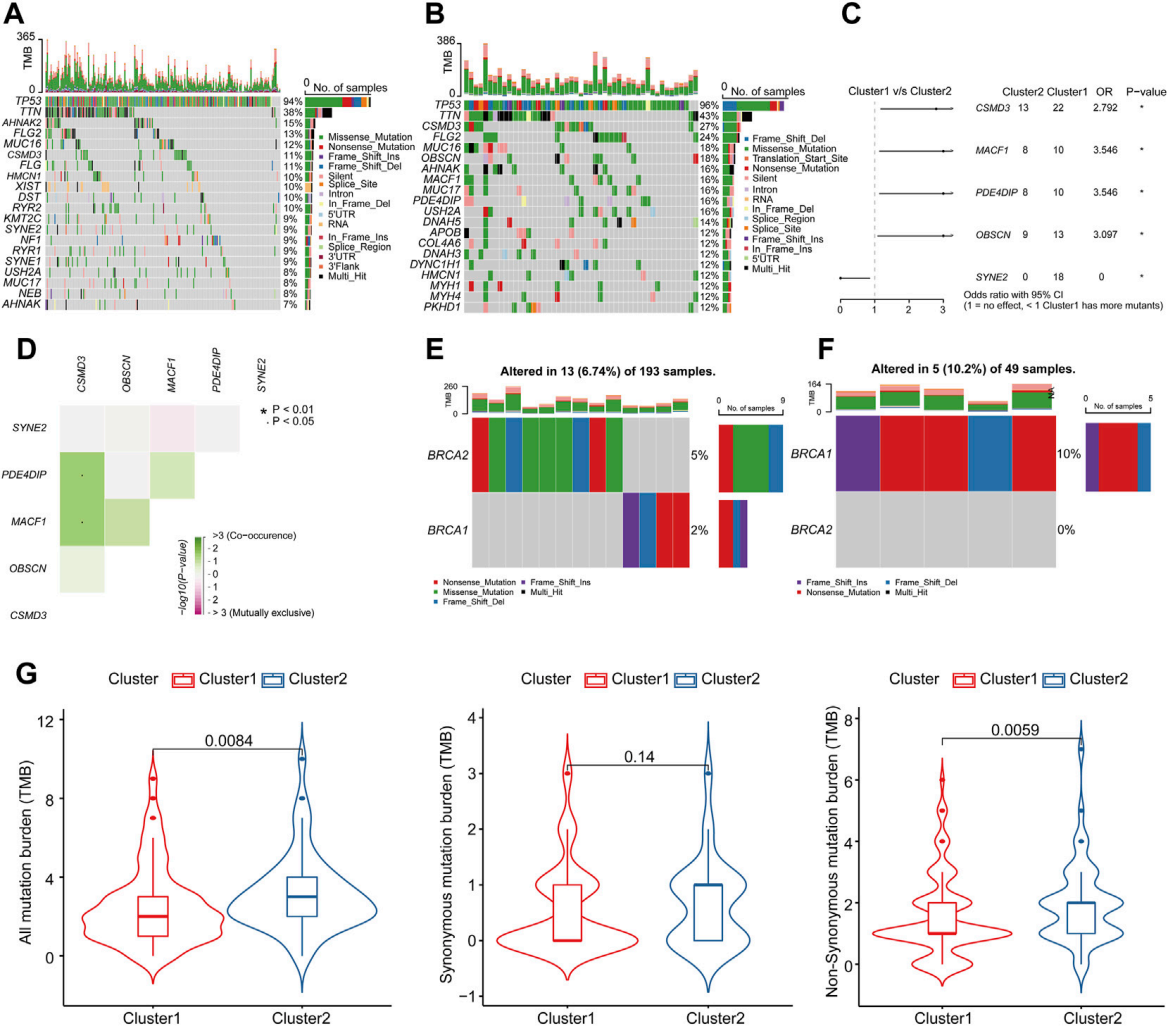
The landscape of mutation profiling in different subtypes of OC. The waterfall plot showed the mutation information of each gene in each sample of **(A)** Cluster 1 and **(B)** Cluster 2. The top panel exhibited the number of variants and the bottom panel showed various mutation types. **(C)** The forest plot described the subgroup analysis of significantly mutated genes. *p < 0.05, and **p < 0.01 determined by one-sided z test and two-sided chi-squared test. **(D)** Mutually co-occurring and exclusive associations across mutated genes in OC were displayed as a triangular matrix. Green and pink indicated a tendency toward co-occurrence and exclusiveness, respectively. The waterfall plot showed the mutation information of BRCA1/2 in each sample of **(E)** Cluster 1 and **(F)** Cluster 2. **(G)** The violin plots showed the TMB differences in different subtypes of OC.

We next explored differences in terms of mutational landscapes between two clusters and the mutation profiles of OC patients were summarized and visualized using the “maftools” R package. The detailed mutation information in each sample was exhibited in a waterfall plot, where different mutation types were presented with various color annotations at the bottom (Figures 5A,B). A horizontal histogram revealed the genes with higher mutation frequency in two OC clusters, respectively. The top 10 mutated signature in Cluster 1 were TP53 (94%), TTN (38%), AHNAK2 (15%), FLG2 (13%), MUC16 (12%), CSMD3 (11%), FLG (11%), HMCN1 (10%), XIST (10%), and DST (10%), while the top 10 mutated signature in Cluster 2 were TP53 (96%), TTN (43%), CSMD3 (27%), FLG2 (24%), MUC16 (18%), OBSCN (18%), AHNAK (16%), MACF1 (16%), MUC17 (16%), and PDE4DIP (16%). Considering the evidence of a correlation between increased immune cell infiltrates and BRCA1/2 mutation was found (McAlpine et al., 2012), we also compared the mutated signature of BRCA1/2 in cluster 1 (Figure 5E) and cluster 2 (Figure 5F), respectively, which presented a slight alteration of BRCA1/2 in cluster 1 (6.74%) and in cluster 2 (10.2%). To get more intuition on the mutational differences, significantly mutated genes between two clusters were presented in Figure 5C. Four genes had dramatically higher mutation frequencies in Cluster 1, including CSMD3, MACF1, PDE4DIP, and OBSCN, while SYNE2 had a higher mutation frequency in Cluster 2. Meanwhile, the presence of significantly co-occurring associations was shown in Figure 5D and Supplementary Table S2, where CSMD3 had the highest correlation with MACF1 and PDE4DIP in their mutation pattern. It has been reported that TMB is closely related to immunotherapeutic response (Snyder et al., 2014; Rizvi et al., 2015). TMB scores of different mutation types were then calculated based on TCGA-OV somatic mutation data and compared between clusters. The results showed that all mutation burdens as well as non-synonymous mutation burdens were significantly higher in Cluster 2 (Figure 5G). In summary, we believe that patients in Cluster 2 had a better effect of immune activation and were more susceptible to immunotherapy.

### The Assessment of Prognostic Value for CD8^+^ T Cell Related Genes

**FIGURE 6:**
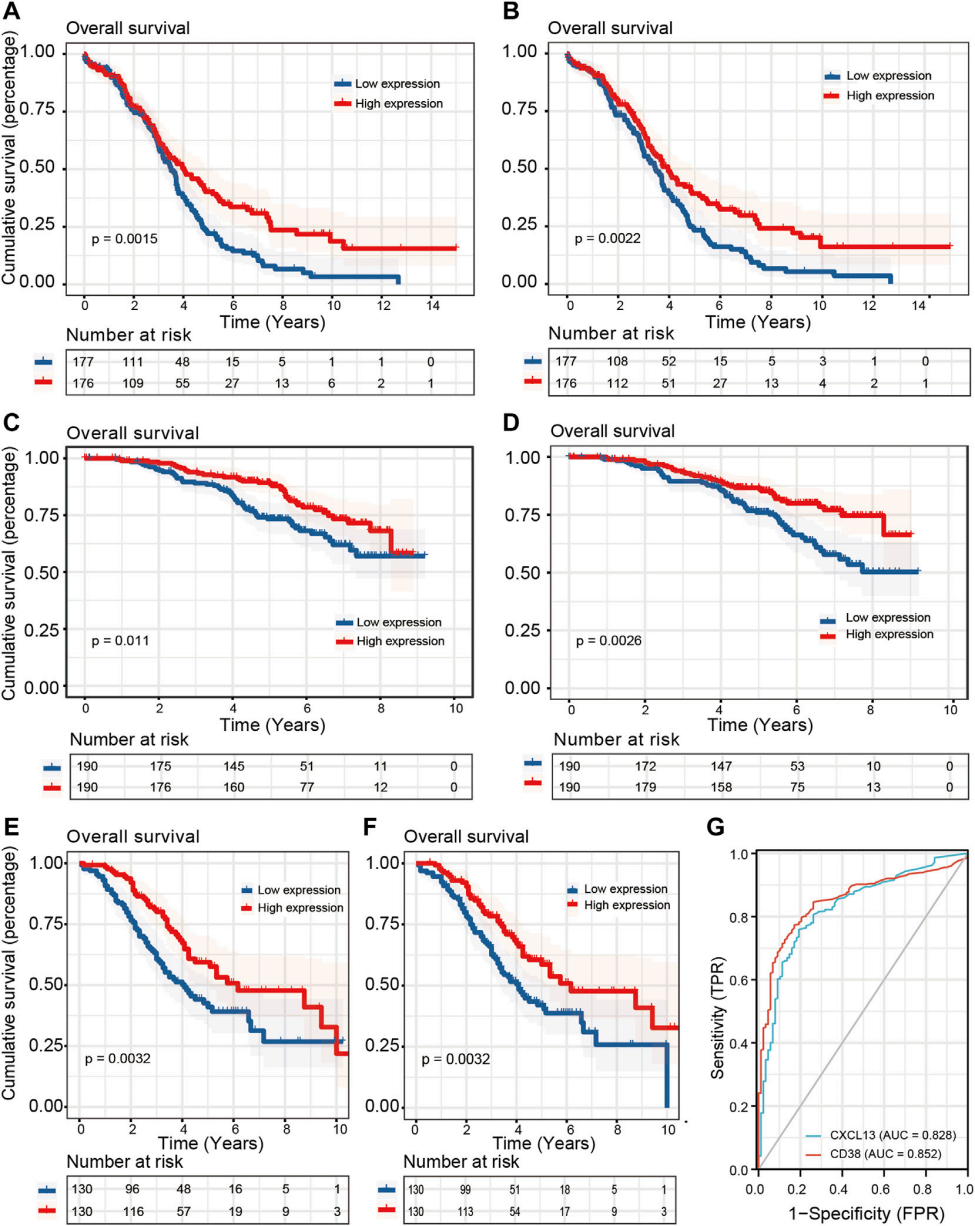
Prognostic value and diagnostic capacity of CD38 and CXCL13 in three OC cohorts. Kaplan-Meier curves were employed to show the correlation between CD38/CXCL13 expression and OS in **(A,B)** TCGA-OV, **(C,D)** GSE140082, and **(E,F)** GSE32062 cohorts. **(G)** The ROC curve was employed to evaluate the diagnostic capacity of CD38 and CXCL13 in OC.

To screen out core essential genes for CD8^+^ T cell-related genes, univariate COX proportional hazards regression analysis and K-M survival analysis were implemented to evaluate the prognostic ability of 313 candidate genes from the WGCNA analysis (Figure 1D). A total of 35 significant prognostic genes were identified by the univariate COX regression analyses, which were then subjected to the K-M survival analyses to get the last 11 genes with survival prognosis in the TCGA-OV cohort. Next, GSE140082 and GSE32062 were used to perform external validation. After conducting univariate COX regression and K-M survival analyses on these 11 genes within two GEO datasets, CD38 and CXCL13 were revealed to have statistical differences among three cohorts (Figures 6A–F; Table 1). We went on to assay the clinical significance of CD38 and CXCL13 and plotted ROC curves. The AUC values for both genes were more than 0.8 (CD38: 0.852, CXCL13: 0.828), indicating a promising predictive performance (Figure 6G). The survival curves of the remaining 9 genes in TCGA-OV, GSE140082, and GSE32062 cohorts were shown in Supplementary Figures S9–S11. We also applied ssGSEA to assess immune infiltrates in the TCGA-OV cohort and analyze their correlation with CD38 and CXCL13 expression (Supplementary Figure S12). Not surprisingly, CD38 and CXCL13 were both highly correlated with T cells and other effector cells with tumor-killing abilities, which was consistent with their better prognosis.

**TABLE 1:** Univariate COX regression and K-M survival analyses of 11 CD8^+^ T cell related genes in TCGA-OV, GSE140082, and GSE32062 cohorts.

Cohort	COX regression					Kaplan-Meier
	Gene	HR	P-value	Lower	Upper	P-value
TCGA-OV Cohort	BATF2	0.985	0.032	0.972	0.999	0.003
	CCR7	0.960	0.043	0.922	0.999	0.006
	**CD38**	**0.962**	**0.038**	**0.927**	**0.998**	**0.001**
	CD3G	0.929	0.014	0.877	0.985	0.017
	CD40LG	0.817	0.003	0.715	0.933	0.045
	CLEC5A	1.020	0.025	1.002	1.037	0.003
	**CXCL13**	**0.994**	**0.024**	**0.989**	**0.999**	**0.002**
	CXCL9	0.997	0.007	0.995	0.999	0.039
	ETV7	0.988	0.047	0.975	1.000	0.037
	HLA-DOB	0.965	0.001	0.945	0.986	0.001
	HLA-F	0.994	0.023	0.989	0.999	0.048
GSE140082 Cohort	BATF2	0.977	0.733	0.856	1.116	0.875
	CCR7	0.929	0.149	0.841	1.027	0.156
	**CD38**	**0.848**	**0.007**	**0.752**	**0.956**	**0.011**
	CD3G	0.836	0.016	0.724	0.967	0.096
	CD40LG	0.849	0.368	0.595	1.212	0.645
	CLEC5A	0.995	0.955	0.839	1.181	0.891
	**CXCL13**	**0.777**	**0.002**	**0.662**	**0.912**	**0.003**
	CXCL9	0.873	0.063	0.756	1.008	0.346
	ETV7	0.858	0.136	0.701	1.050	0.037
	HLA-DOB	0.887	0.043	0.790	0.996	0.027
	HLA-F	0.880	0.175	0.731	1.059	0.318
GSE32062	BATF2	0.894	0.090	0.786	1.018	0.105
	CCR7	0.871	0.015	0.778	0.974	0.077
	**CD38**	**0.861**	**0.002**	**0.784**	**0.945**	**0.003**
	CD3G	0.883	0.023	0.793	0.983	0.071
	CD40LG	0.789	0.103	0.593	1.049	0.365
	CLEC5A	0.826	0.014	0.709	0.961	0.056
	**CXCL13**	**0.905**	**0.008**	**0.840**	**0.975**	**0.003**
	CXCL9	0.895	0.003	0.832	0.962	0.007
	ETV7	0.867	0.009	0.779	0.965	0.053
	HLA-DOB	0.896	0.165	0.767	1.046	0.283
	HLA-F	0.778	0.001	0.668	0.906	0.014

Bold for highlights of the results of CD38 and CXCL13.

### Analysis of the Immunotherapeutic Value of CD38 and CXCL13

**FIGURE 7:**
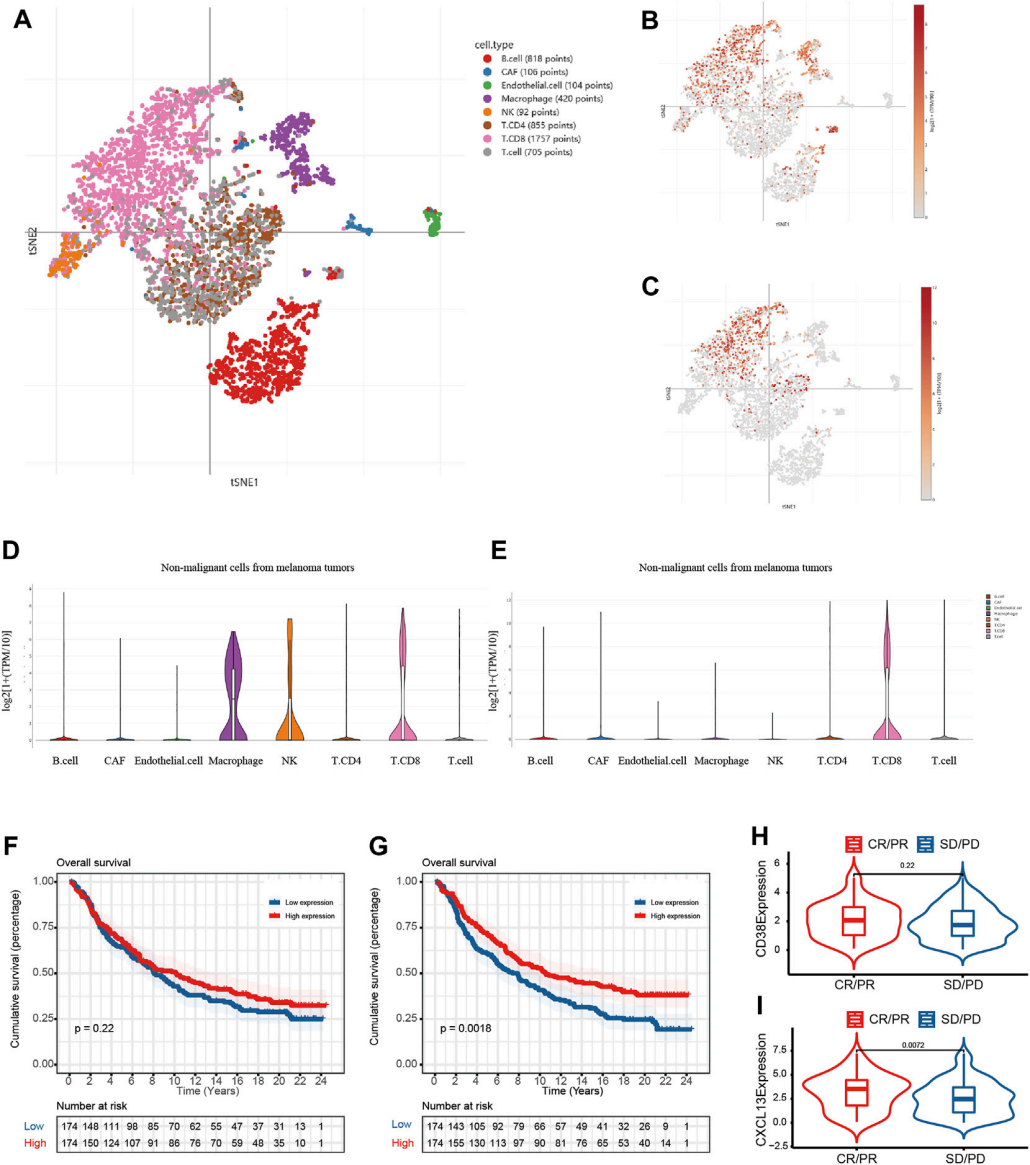
The potential prognostic value of CD38 and CXCL13 in OC patients with immunotherapy. **(A)** tSNE plot of various cell types in the GSE115978 cohort; **(B,C)** The expression of CD38 in multiple cells; **(D,E)** The expression of CXCL13 in multiple cells; Kaplan-Meier curves showed the prognosis of **(F)** CD38 and **(G)** CXCL13 in the IMvigor210 cohort; The violin plots showed the difference of **(H)** CD38 and **(I)** CXCL13 expression in the CR/PR and SD/PD groups. CR, complete response; PR, partial response; SD, stable disease; PD, progressive disease.

We next involved two immunotherapy treated cohorts (GSE115978 single-cell cohort and IMvigor 210 cohort) to determine whether CD38 and CXCL13 could be served as emerging predictive biomarkers for immune therapy. tSNE plot of various cell types in the GSE115978 cohort were provided in Figure 7A. As shown in Figures 7B,C, CD38 was significantly elevated not only in CD8^+^ T cells, but also in macrophages, NK cells, and CD4^+^ T cells. As such, the expression of CXCL13 in various immune cell types was provided in Figures 7D,E, revealing a higher expression not only in CD8^+^ T cells but also in B cells, and cancer-associated fibroblasts (CAF). We also performed survival analysis in the IMvigor210 cohort when mRNA expression data and clinical data were combined. The results showed that there was no significant relationship between CD38 expression and the prognosis, while low CXCL13 expression had a significant correlation with poor prognosis (Figures 7F,G). In addition, no statistically significant difference in CD38 expression was observed between patients with CR/PR and SD/PD (CR, complete response; PR, partial response; SD, stable disease; PD, progressive disease), while the CR/PR group had a significantly higher expression of CXCL13 than the SD/PD group (Figures 7H,I). The results above illustrated that CXCL13 was a prognostic biomarker in OC patients with immunotherapy, showing its potential ability as an immunotherapeutic target.

## Discussion

OC remains the most lethal gynecologic malignancy worldwide and more treatment options are on the horizon (Kuroki and Guntupalli, 2020). The utilization of immunotherapy has emerged as a potential new frontier in the treatment of OC; however, the therapeutic effect is not always satisfactory. Cytotoxic CD8^+^ T cell plays a pivotal role in antitumor response and its infiltration is a precondition for tumor immunity in the TME. In this presented study, we used the CIBERSORT algorithm to generate computational estimates for the relative proportion of CD8^+^ T cells in the TCGA-OV and GSE140082 cohorts. WGCNA analysis was then performed to identify 313 CD8^+^ T cell-related genes in OC, based on which two prognostic clusters from the TCGA-OV cohort were uncovered. Patients from the Cluster 1 group exhibited worse prognosis, decreased immune score, lower abundance of CD8^+^ T cells, less sensitivity to immunotherapy, and lower TMB. Functional enrichment analyses suggested that DEGs between two clusters were mainly enriched in T cell activation and chemokine receptor binding. CSMD3, MACF1, PDE4DIP, and OBSCN were more frequently mutated in Cluster 1, while SYNE2 was more frequently mutated in Cluster 2. We further explored the key genes involved in the CD8^+^ T cell-mediated immune response and CD38 and CXCL13 were confirmed to contain the prognostic and diagnostic values. More importantly, CXCL13 showed better performance in two immunotherapy treated cohorts, highlighting its potential therapeutic value in OC.

We observed a significant difference in TME between the two clusters via the ESTIMATE and MCPcounter tools in the TCGA-OV cohort. TME has been proved to participate in the initiation and progression of tumorigenesis, thus attracting a colossal number of studies. In OC, immune cell populations, including cytotoxic T and B lymphocytes, NK cells, Tregs, etc., have a substantial importance in the treatment (Santoiemma et al., 2016; Rodriguez et al., 2018a; Yang et al., 2020). Meanwhile, other components including fibroblasts and adipocytes might also influence the efficacy of standard treatments or immunotherapies (Rodriguez et al., 2018b). In our study, tumor-infiltrating lymphocytes (TILs) such as CD8^+^ T cells, B lymphocytes, as well as innate immune cells such as NK cells, and myeloid dendritic cells were more enriched in the Cluster 2 group. The prognostic significance of TILs in OC has been confirmed in a meta-analysis with 1815 patients from Hwang’s study in 2012 (Rodriguez et al., 2018b). Intraepithelial TILs were presented as a robust predictor of clinical outcome in OC regardless of the tumor grade, stage, or histologic subtype and a lack of TILs was significantly associated with worse survival among patients. Stromal and intraepithelial B lymphocytes characterized by the production of tumor-specific IgG Subclasses (IGGS) were also reported to have a positive role in patients with high-grade serous OC (Montfort et al., 2017). Meanwhile, the strong B-cell memory response could be enhanced by chemotherapy. Similar results could also be observed in NK cells and dendritic cells (Bamias et al., 2007; Tsiatas et al., 2009; Okła et al., 2016), which was consistent with our finding that the Cluster 2 group with more TILs showed a better prognosis. Chemokine-associated responses to antitumor immunity were found in various TMEs, further illustrating the essentiality to establish predictive biomarkers of TME to enhance the immunotherapy benefit in OC (Rainczuk et al., 2012; Viola et al., 2012; Duan et al., 2018). Not surprisingly, the better-performed Cluster 2 had significantly elevated chemokines with receptors and immune activators. At the same time, the assessment of immunotherapy by the ssGSEA method also revealed that Cluster 2 had significantly elevated scores. Enrichment analyses showed that DEGs in Cluster 2 were mainly enriched in natural killer cell-mediated cytotoxicity, T cell receptor signaling pathway, and Th cell differentiation. The above results indicated that patients classified based on CD8^+^ T cells had distinct prognoses, TME, and responses to immunotherapy.

The mutation frequencies were distinctly different between the two subtypes. Lu et al. (2021)’s study pointed out that the overall survival of OC patients with CSMD3 mutation was inferior to those with wild-type CSMD3 and CSMD3 mutation was highly correlated with increased TMB. Cheasley et al. (2021) performed a comprehensive genomic analysis of low-grade serous ovarian carcinoma patients and found that MACF1 had an 11% mutation frequency as a putative novel driver gene, which could be translated into an improved therapeutic path. Er et al. (2016) identified PDE4DIP as a recurrently mutated gene in endometriosis-associated OC and OBSCN was found to mutate on at least two sites in OC from Zhang’s study (Zhang et al., 2019). In our study, CSMD3, MACF1, PDE4DIP, and OBSCN were more frequently mutated in Cluster 1 with a worse prognosis, which was consistent with published literature. SYNE2 was more frequently mutated in Cluster 2 and has been observed in Emery-Dreifuss muscular dystrophy (Lee et al., 2020) and retinal defects (Maddox et al., 2015). However, the specific biological significance of SYNE2 in OC remains to be determined. The performance of TMB has been verified in various cancers to predict responses to immunotherapy, such as lung cancer (Rizvi et al., 2015) and melanoma (Snyder et al., 2014). The previous study has reported that a higher TMB was significantly correlated with better prognosis and lower clinical stages and tumor-free status (Fan et al., 2020). The infiltrating level of immune cells was also significantly elevated in the high-TMB group than in the low-TMB group. In our study, the Cluster 2 group showed a significantly higher level of not only all mutation burden but also non-synonymous mutation burden, with the remarkable consistency of the infiltrating TILs and better response to immunotherapy.

We next explored the key CD8^+^ T cell-related genes, which may participate in the anti-tumor immune response in OC. After the conduction of COX regression analyses combined with K-M survival analyses in three cohorts, CD38 and CXCL13 were screened out. Meanwhile, they both showed the diagnostic and predictive value and were highly correlated with immune infiltrates. CD38 is a non-lineage restricted, type II transmembrane glycoprotein with ectoenzymatic functions, which participates in the mediation of NAD^+^ homeostasis (Hogan et al., 2019). Recent studies have described CD38 as a surface marker for lymphocytes and its involvement in CD8^+^ T cell suppression in TME led to the resistance to PD-1/PD-L1 blockade therapy (Chen et al., 2018; Hogan et al., 2019). In Zhu’s research, CD38 was found to be positively correlated with prognosis and immune cell infiltration in the microenvironment of OC and contributed to the antitumor immunity, which was consistent with our finding (Zhu et al., 2020). Although CD38 and CXCL13 both had prognostic values in three OC cohorts, only CXCL13 was shown to perform well after including two immunotherapy cohorts. CXC-chemokine ligand 13 (CXCL13) uniquely binds to the chemokine receptor CXCR5, which is strongly expressed on B cells, follicular helper T (Tfh) cells, and follicular cytotoxic T (Tfc) cells (He et al., 2016; Im et al., 2016). Thus, CXCL13 preferentially promotes the migration of B lymphocytes to the site of chronic inflammation to orchestrate humoral and adaptive immune responses (Gu-Trantien et al., 2013; Tirosh et al., 2016). Yang et al. (2021) found that CXCL13 was colocalized with tertiary lymphoid structures and played a pivotal role in shaping the antitumor microenvironment by facilitating the maintenance of CXCR5^+^CD8^+^ T cells. More importantly, their research further supported a clinical investigation for a combination of CXCL13 and anti-PD-1 therapy in human high-grade serous OC tumors and murine models. CXCL13 was able to increase the infiltration of cytotoxic CD8^+^ T cells, thus retarding tumor growth in a CD8^+^ T cell-dependent manner. Consistently, our work also shed light on the therapeutic value of CXCL13 as the CD8^+^ T cell-related marker.

In summary, this study was the first attempt to use the WGCNA and CIBERSORT algorithms to identify CD8^+^ T cell-related genes of OC. Two prognostically and clinically relevant clusters were then identified to exhibit distinct TME and TMB. Through multiple verifications, CXCL13 was identified as a potential biomarker and therapeutic target for OC immunotherapy. However, our study has several limitations. Considering the limited sample data, a multicenter prospective cohort study should be taken to verify the results and the specific mechanism of CXCL13 in OC requires further investigation.

## Data Availability

The datasets presented in this study can be found in online repositories. The names of the repository/repositories and accession number(s) can be found in the article/Supplementary Material.

## Supplementary Material

Supplementary Figure S1 The relative abundance of 22 distinct immune cell types in the **(A)** TCGA-OV cohort and GSE140082 cohort.

Supplementary Figure S2 Weighted gene co-expression network analysis in the TCGA-OV cohort. **(A)** Sample dendrogram with trait heatmap. **(B)** Dendrogram of differentially expressed genes clustered based on different metrics.

Supplementary Figure S3 Weighted gene co-expression network analysis in the GSE140082 cohort. **(A)** Sample dendrogram with trait heatmap. **(B)** Analysis of the scale-free fit index and the average connectivity of various soft threshold power. **(C)** Dendrogram of differentially expressed genes clustered based on different metrics.

Supplementary Figure S4 Consensus maps showed the correlation profiling of OC patients derived from 2 to 10 sub-consensuses.

Supplementary Figure S5 Verification of the subclass distribution. **(A)** PCA and **(B)** t-SNE analyses supported the classification of OC into two subtypes.

Supplementary Figure S6 The violin plots showed **(A)** the immune score, **(B)** the stromal score, **(C)** the ESTIMATE score, and **(D)** the tumor purity in different OC subtypes.

Supplementary Figure S7 The boxplots showed the gene expression (log) of 148 immunomodulators in different OC subtypes: **(A)** chemokine; **(B)** receptor; **(C)** MHC; **(D)** immunostimulatory; **(E)** immunoinhibitory.

Supplementary Figure S8 Functional enrichment analyses of OC clusters. **(A)** GO and **(B)** KEGG pathway analyses of DEGs between CD8+ T cell-based molecular subtypes. Oncological signatures were significantly enriched in **(C)** Cluster 1 and **(D)** Cluster 2 identified by gene set enrichment analysis (GSEA).

Supplementary Figure S9 The survival curves of the remaining 9 genes in the TCGA-OV **(A)** cohort. BATF2; **(B)** CCR7; **(C)** CD3G; **(D)** CD40LG; **(E)** CLEC5A; **(F)** CXCL9; **(G)** ETV7; **(H)** HLA_DOB; **(I)** HLA_F.

Supplementary Figure S10 The survival curves of the remaining 9 genes in the GSE140082 cohort. **(A)** BATF2; **(B)** CCR7; **(C)** CD3G; **(D)** CD40LG; **(E)** CLEC5A; **(F)** CXCL9; **(G)** ETV7; **(H)** HLA_DOB; **(I)** HLA_F.

Supplementary Figure S11 The survival curves of the remaining 9 genes in the GSE32062 **(A)**cohort. BATF2; **(B)** CCR7; **(C)** CD3G; **(D)** CD40LG; **(E)** CLEC5A; **(F)** CXCL9; **(G)** ETV7; **(H)** HLA_DOB; **(I)** HLA_F.

Supplementary Figure S12 The lollipop charts showed the correlation between the immune infiltrates and **(A)**CD38 and **(B)** CXCL13 expression in the TCGA-OV cohort.
