# Supplementary figures and images for: Identification of CD8+ T Cell Related Biomarkers in Ovarian Cancer
Source: Front Genet. 2022 May 27;13:860161. doi: 10.3389/fgene.2022.860161 (PMC9196910; doi:10.3389/fgene.2022.860161)

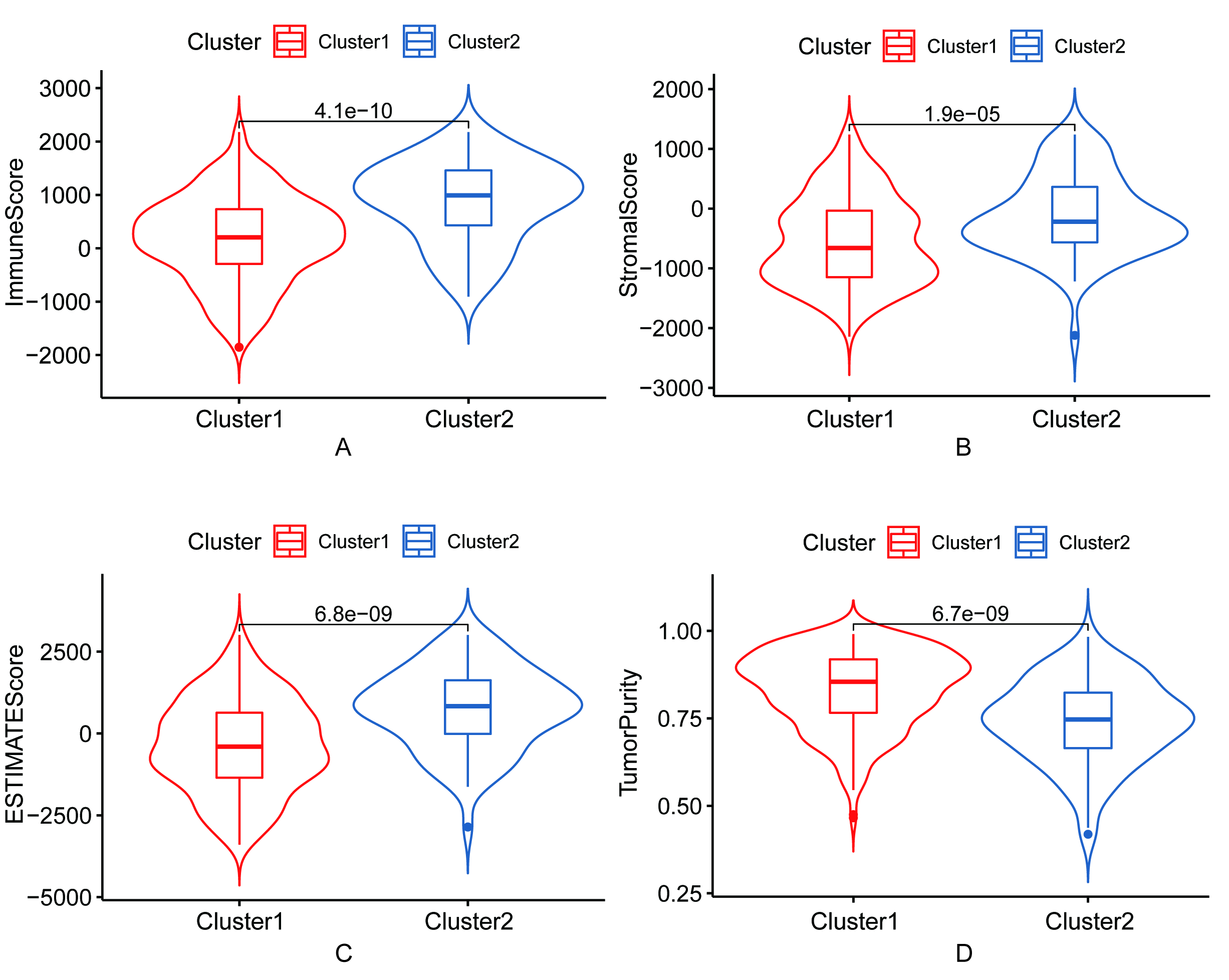

Supplement: Supplementary file 1 [file Image6.TIF]

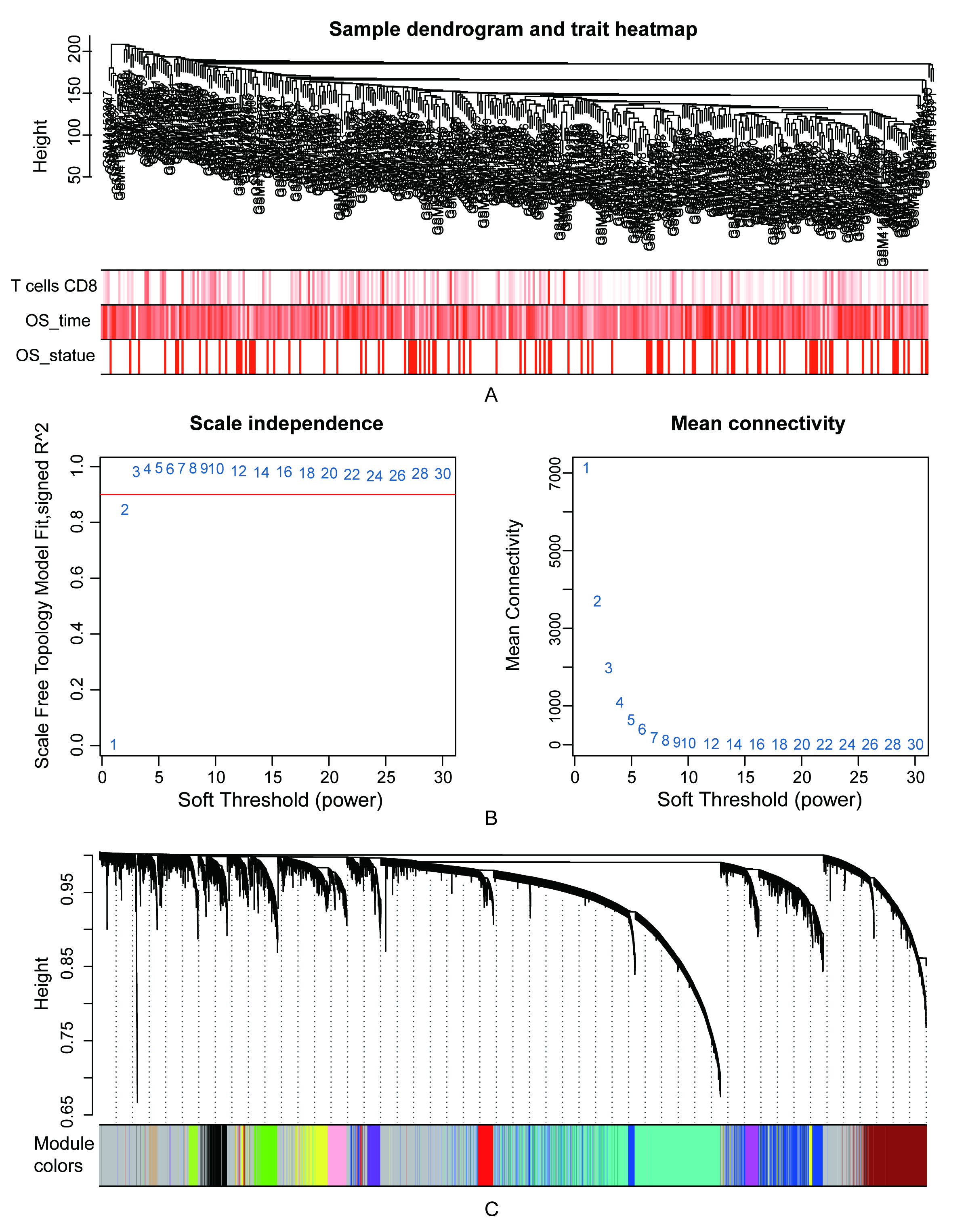

Supplement: Supplementary file 3 [file Image3.TIF]

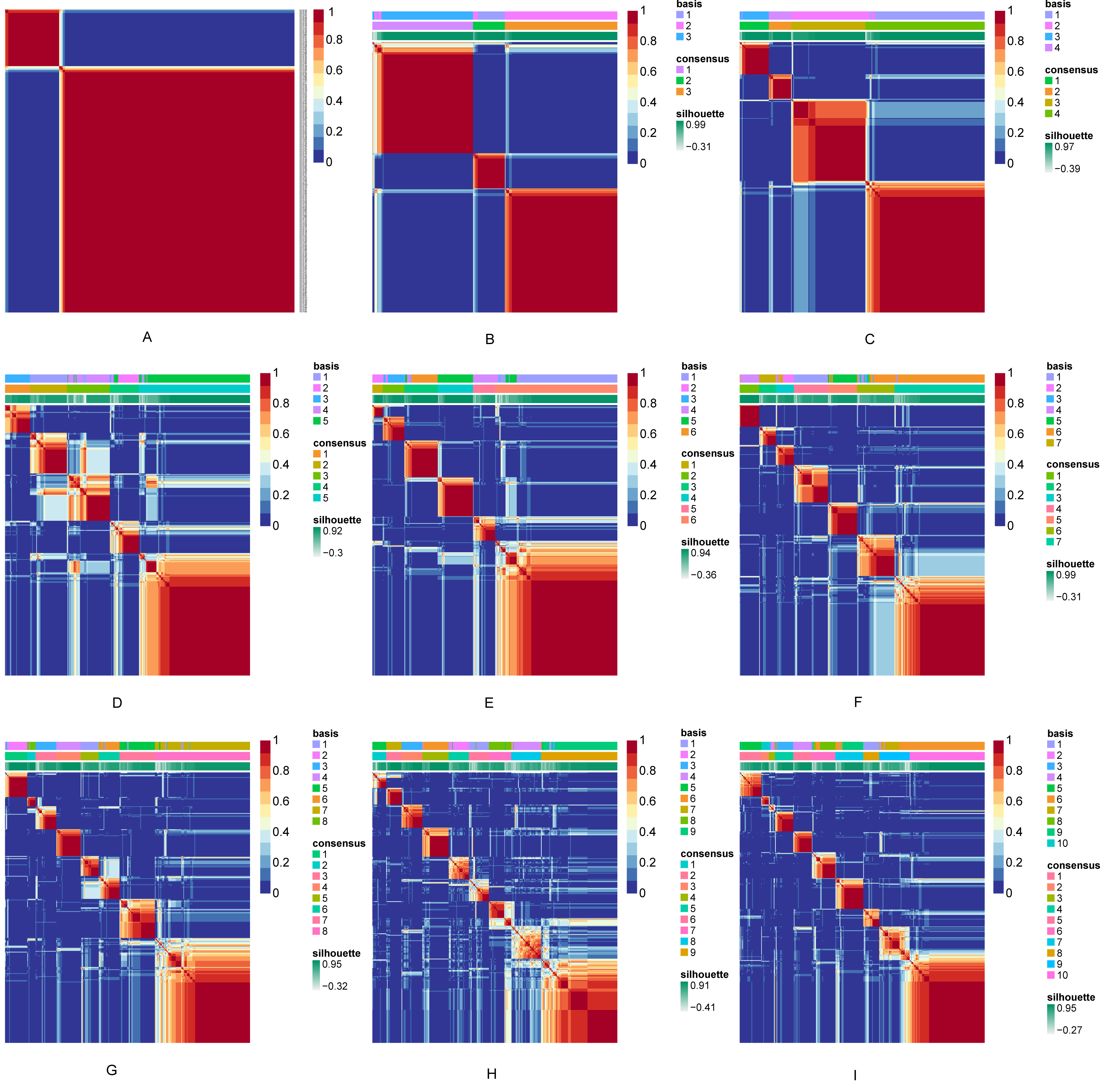

Supplement: Supplementary file 4 [file Image4.TIF]

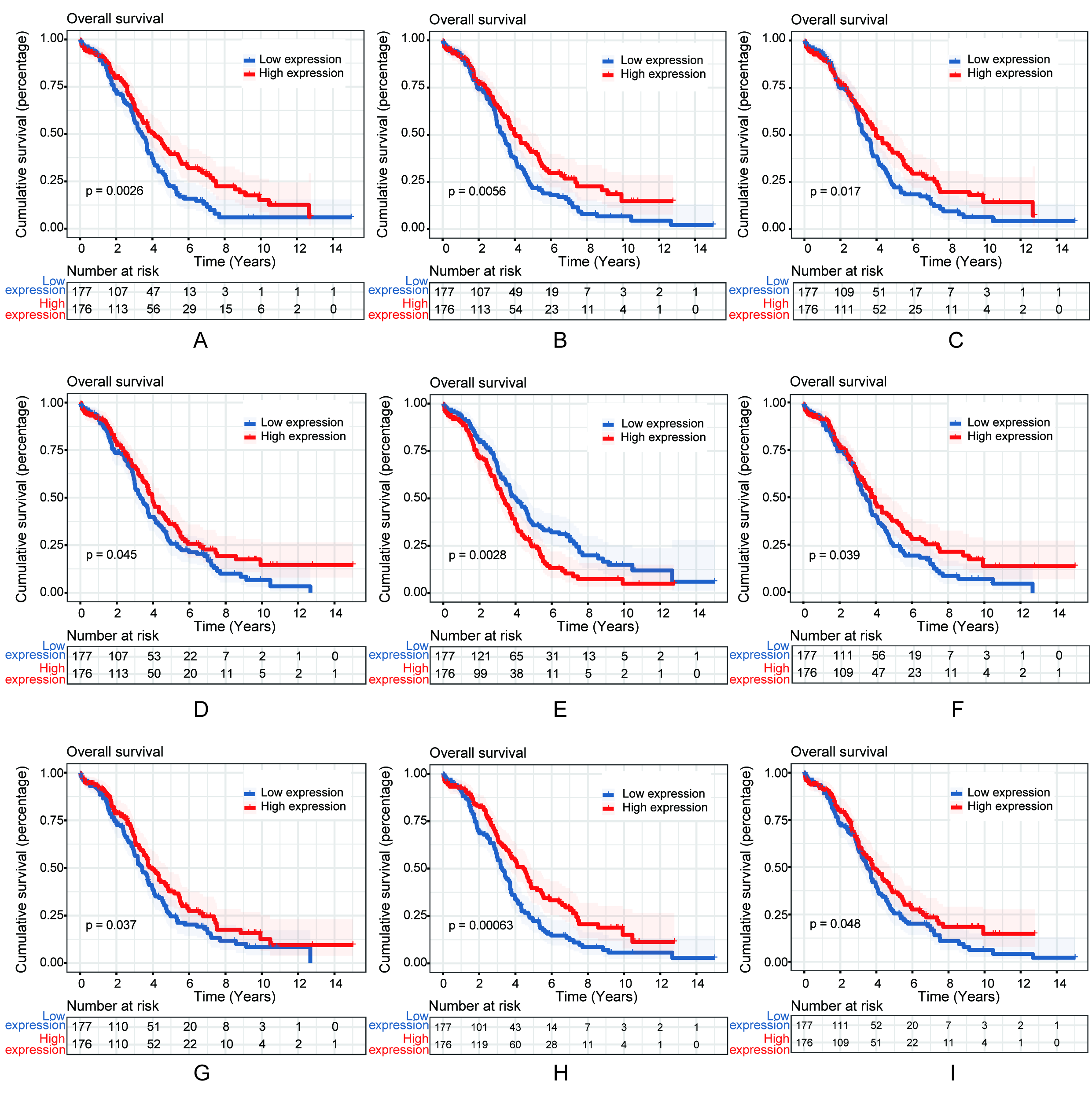

Supplement: Supplementary file 5 [file Image9.TIF]

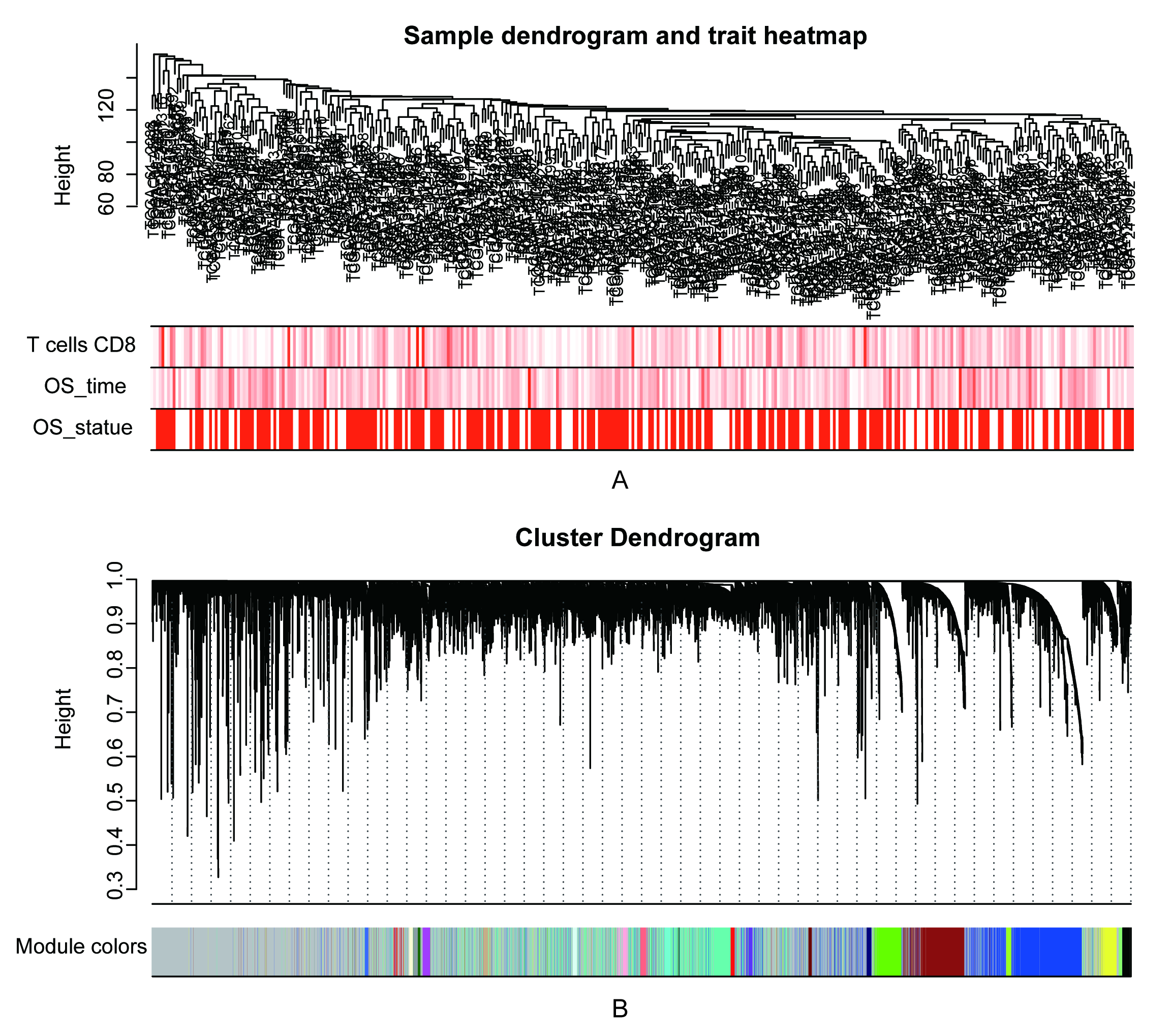

Supplement: Supplementary file 6 [file Image2.TIF]

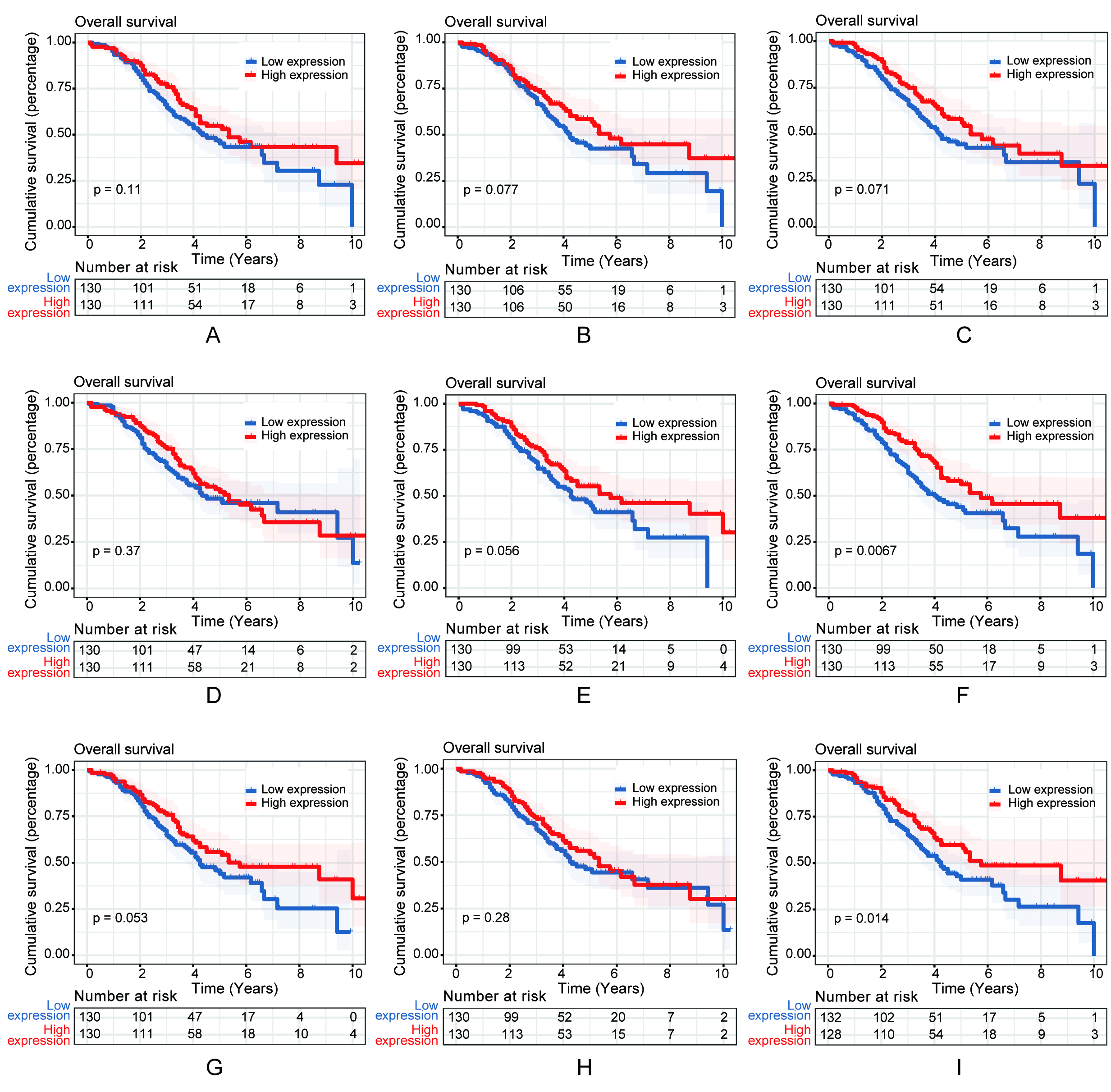

Supplement: Supplementary file 8 [file Image11.TIF]

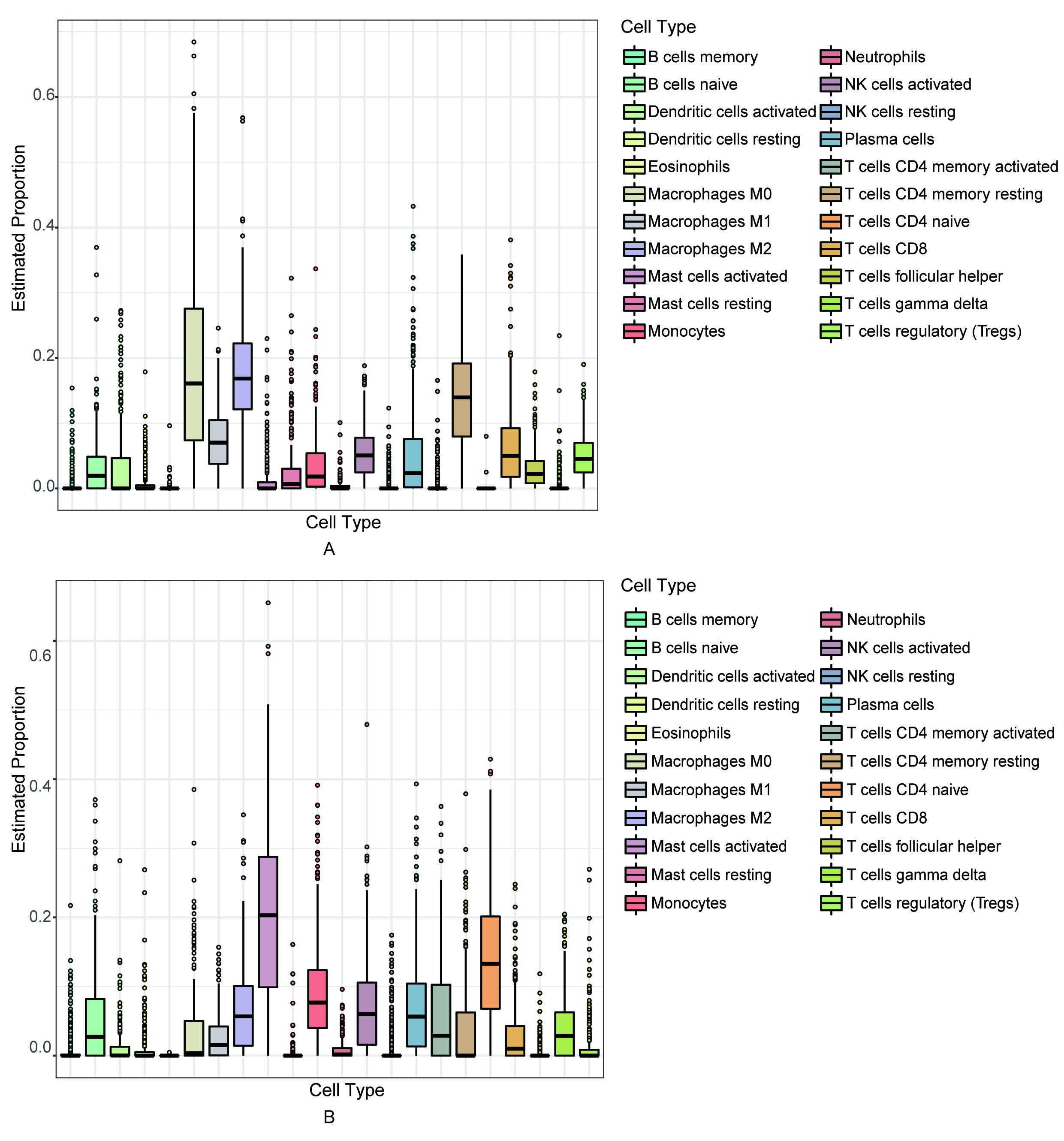

Supplement: Supplementary file 9 [file Image1.TIF]

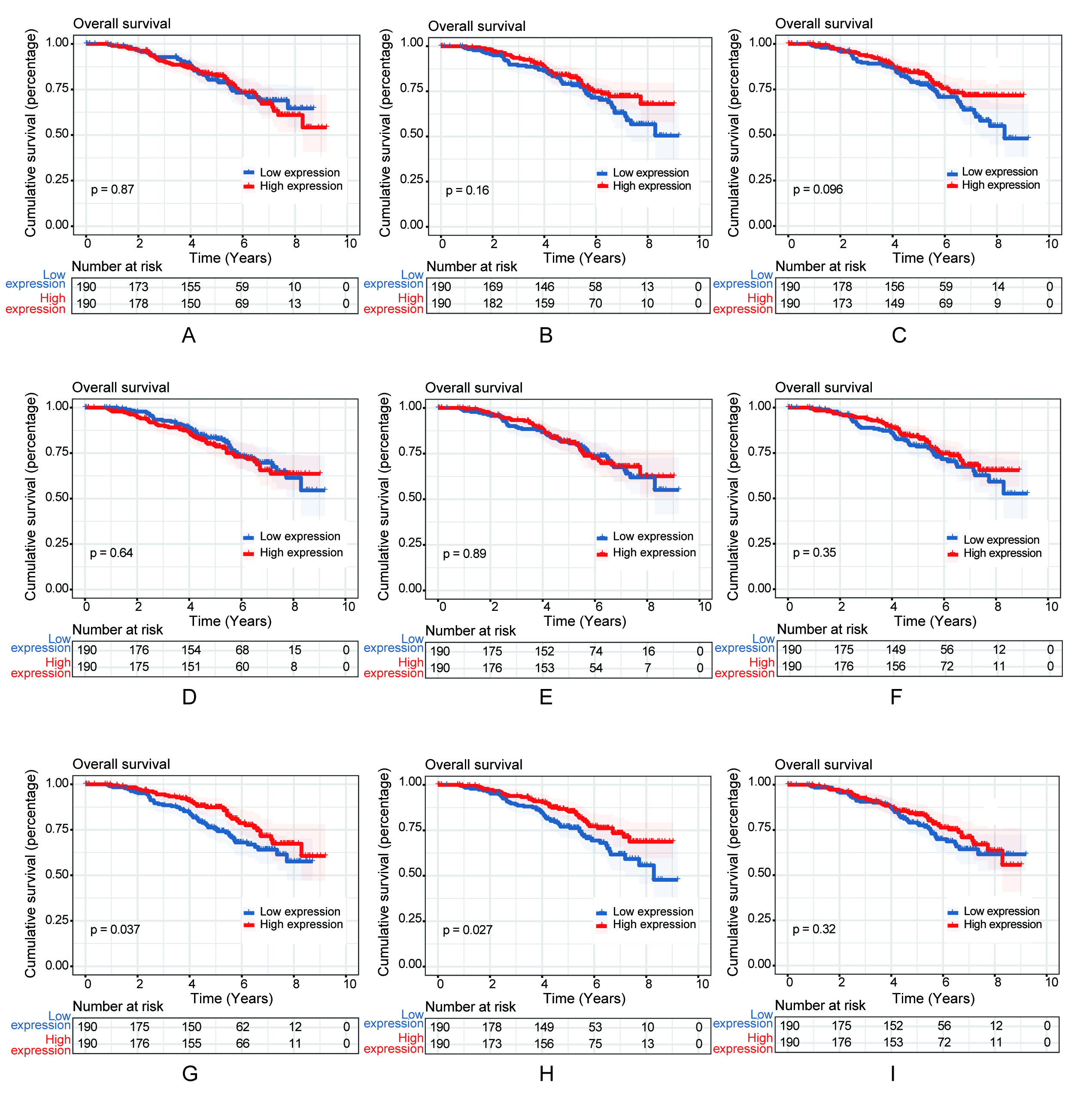

Supplement: Supplementary file 10 [file Image10.TIF]

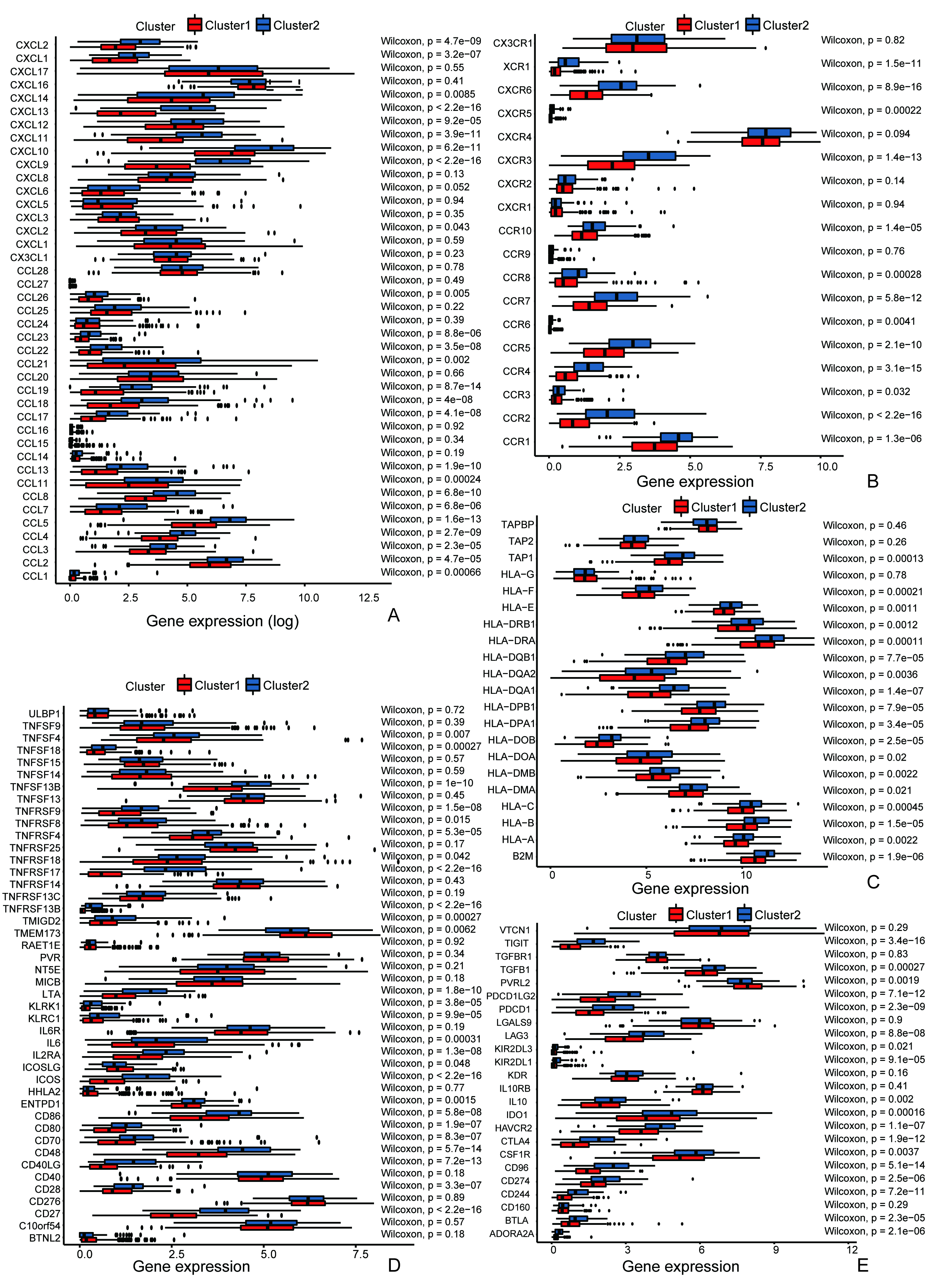

Supplement: Supplementary file 11 [file Image7.TIF]

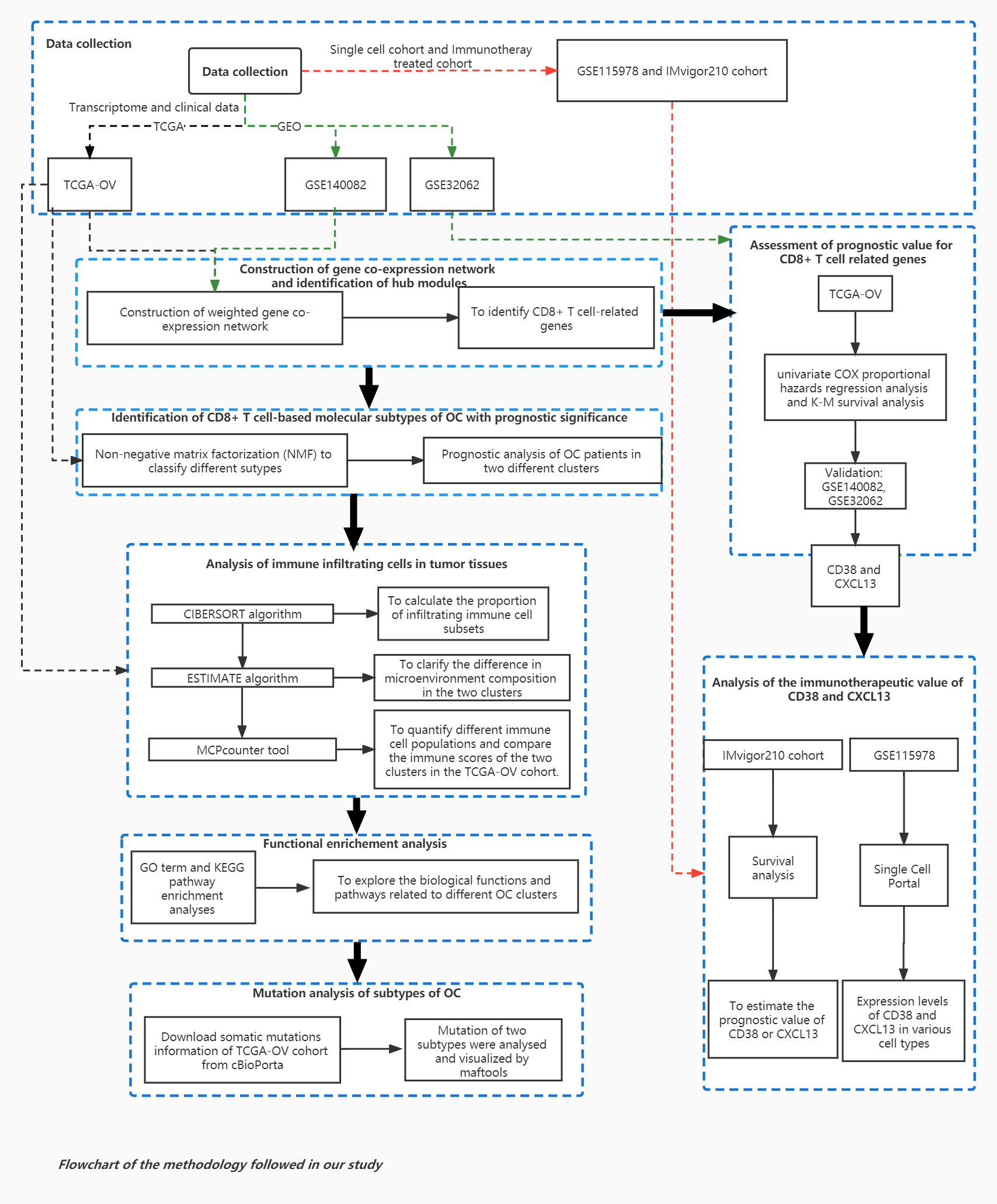

Supplement: Supplementary file 12 [file Image13.JPEG]

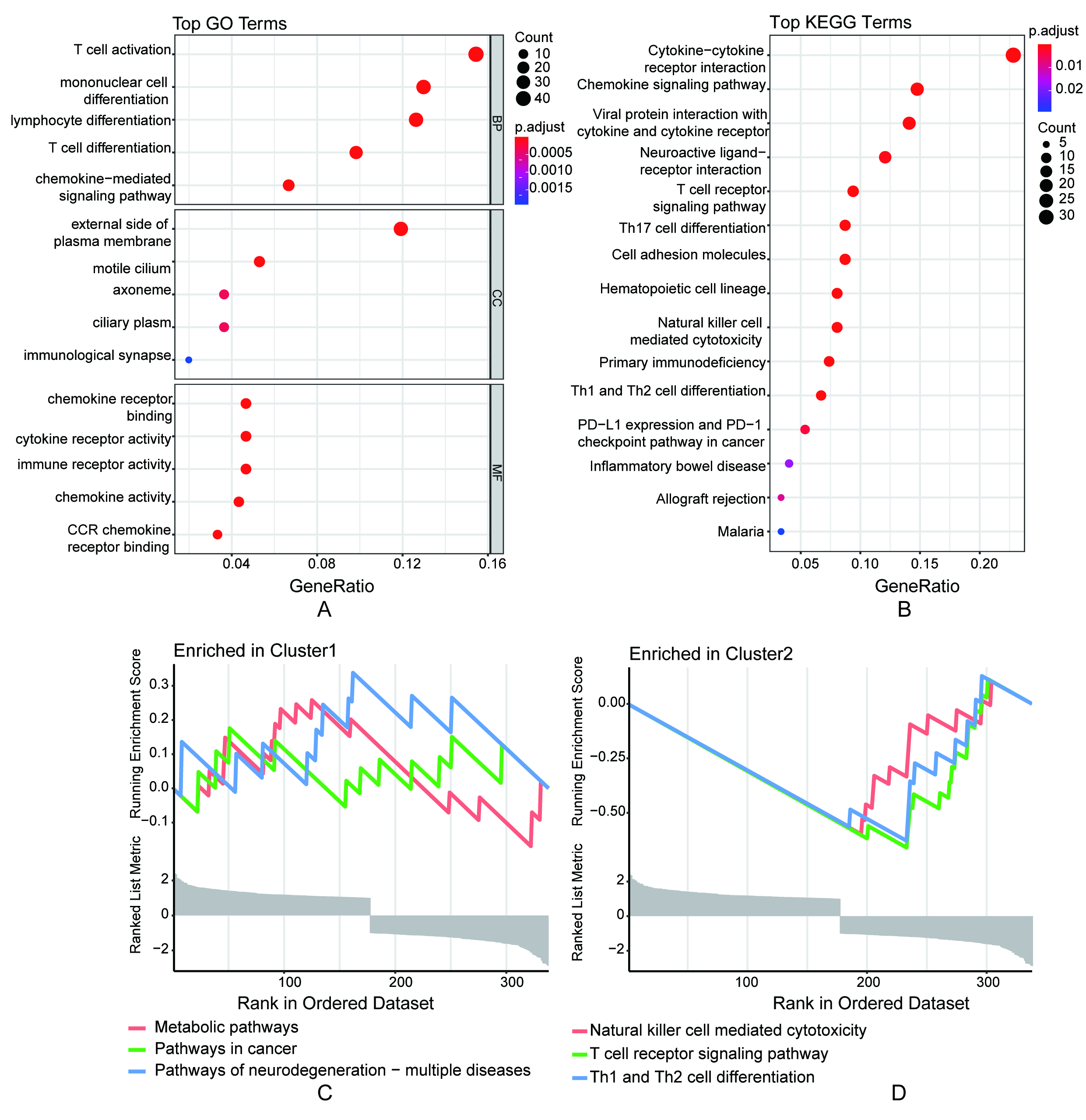

Supplement: Supplementary file 13 [file Image8.TIF]

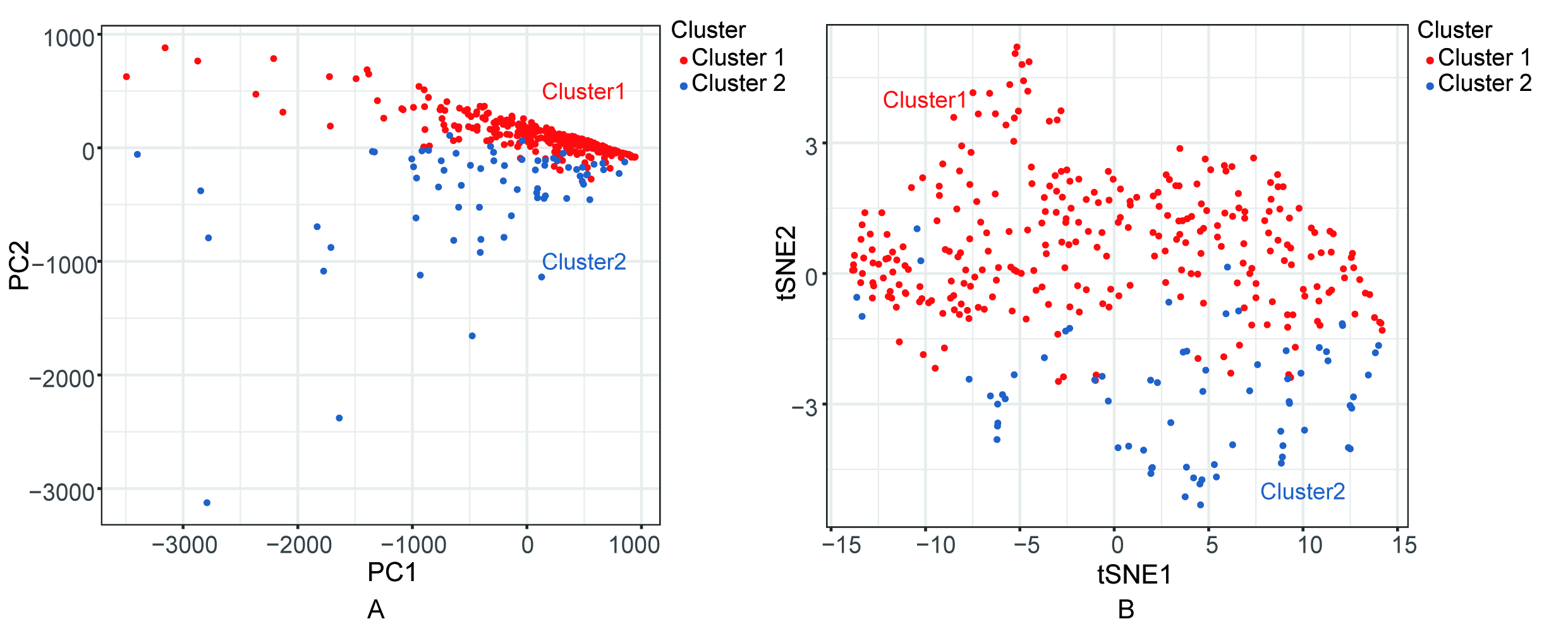

Supplement: Supplementary file 14 [file Image5.TIF]

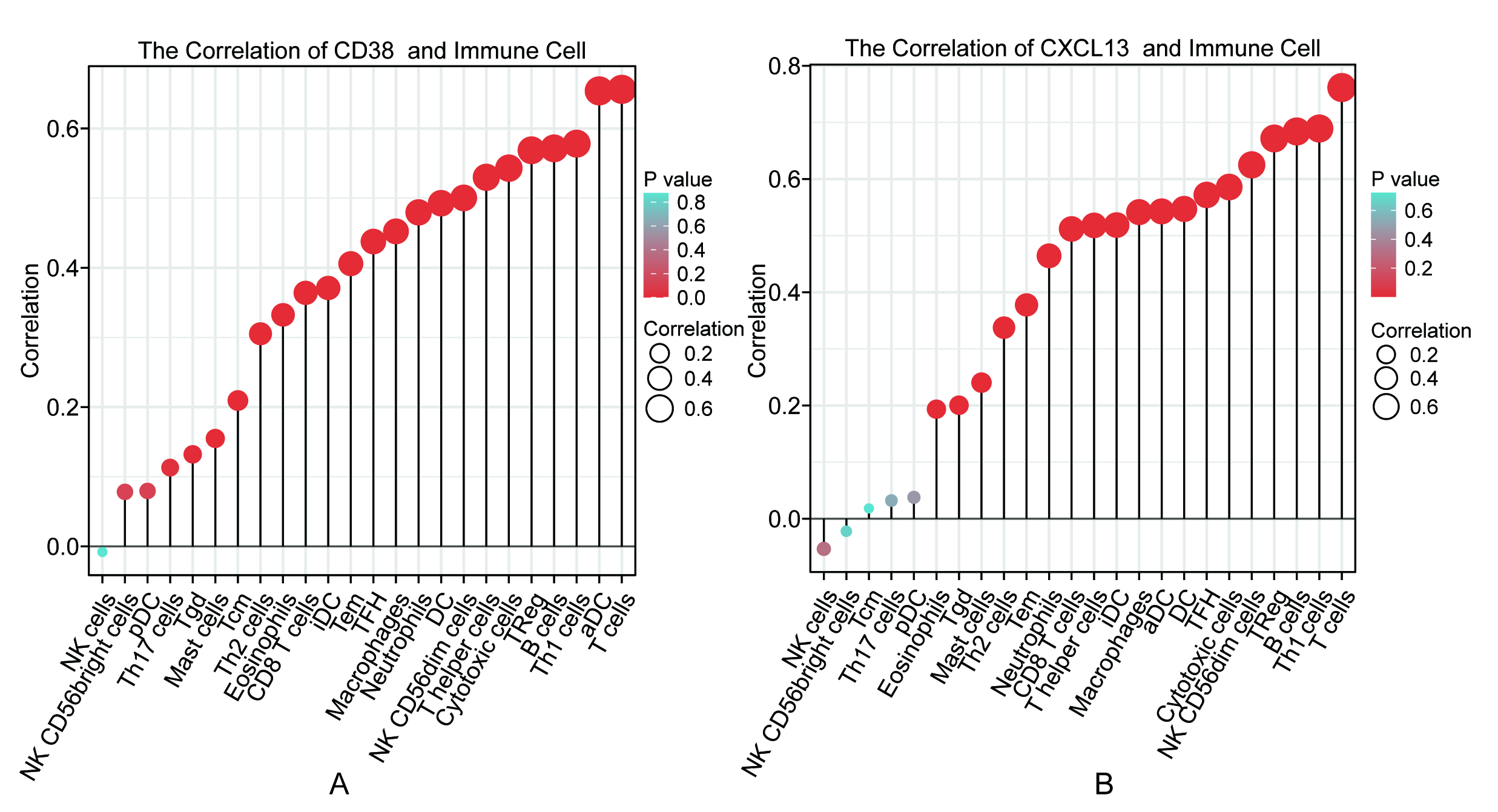

Supplement: Supplementary file 15 [file Image12.TIF]
