# Supplementary material for: Identification of CD8+ T Cell Related Biomarkers in Ovarian Cancer
Source: Front Genet. 2022 May 27;13:860161. doi: 10.3389/fgene.2022.860161 (PMC9196910; doi:10.3389/fgene.2022.860161)
Supplement: Supplementary file 2 [file Table1.DOCX]

**Supplementary materials.**

**Table 1. Information of public data used in this study**

| Data Cohort | Sample (n) | Platform | immunotherapy |
| --- | --- | --- | --- |
| TCGA-OV | 353 | Next-Generation Sequencing |  |
| GSE140082 | 380 | GPL14951 Illumina HumanHT-12 |  |
| GSE32062 | 260 | GPL570/GPL6480 Agilent G4112F |  |
| GSE115978 | 31 | GPL18573 Illumina NextSeq 500 (Single Cell) | Anti CTLA-4, anti PD-1 (Ipilimumab, nivolumab, Tremelimumab, Pembrolizumab, etc.) |
| IMvigor210 | 348 | Next-Generation Sequencing | AntiPD-L1 (atezolizumab) |
