# Supplementary material for: Identification of CD8+ T Cell Related Biomarkers in Ovarian Cancer
Source: Front Genet. 2022 May 27;13:860161. doi: 10.3389/fgene.2022.860161 (PMC9196910; doi:10.3389/fgene.2022.860161)
Supplement: Supplementary file 7 [file Image14.pdf]

Appendix

Figure legends of supplementary figures.

Supplementary Figure S1 | The relative abundance of 22 distinct immune cell types in the (A) TCGA-OV cohort and GSE140082 cohort.

Supplementary Figure S2 | Weighted gene co-expression network analysis in the TCGA-OV cohort. **(A)** Sample dendrogram with trait heatmap. **(B)** Dendrogram of differentially expressed genes clustered based on different metrics.

Supplementary Figure S3 | Weighted gene co-expression network analysis in the GSE140082 cohort. **(A)** Sample dendrogram with trait heatmap. **(B)** Analysis of the scale-free fit index and the average connectivity of various soft threshold power. **(C)** Dendrogram of differentially expressed genes clustered based on different metrics.

Supplementary Figure S4 | Consensus maps showed the correlation profiling of OC patients derived from 2 to 10 sub-consensuses.

Supplementary Figure S5 | Verification of the subclass distribution. **(A)** PCA and **(B)** t-SNE analyses supported the classification of OC into two subtypes.

Supplementary Figure S6 | The violin plots showed **(A)** the immune score, **(B)** the stromal score, **(C)** the ESTIMATE score, and **(D)** the tumor purity in different OC subtypes.

Supplementary Figure S7 | The boxplots showed the gene expression (log) of 148 immunomodulators in different OC subtypes: **(A)** chemokine; **(B)** receptor; **(C)** MHC; **(D)** immunostimulatory; **(E)** immunoinhibitory.

Supplementary Figure S8 | Functional enrichment analyses of OC clusters. **(A)** GO and **(B)** KEGG pathway analyses of DEGs between CD8+ T cell-based molecular subtypes. Oncological signatures were significantly enriched in **(C)** Cluster 1 and **(D)** Cluster 2 identified by gene set enrichment analysis (GSEA).

Supplementary Figure S9 | The survival curves of the remaining 9 genes in the TCGA-OV cohort. **(A)** BATF2; **(B)** CCR7; **(C)** CD3G; **(D)** CD40LG; **(E)** CLEC5A; **(F)** CXCL9; **(G)** ETV7; **(H)** HLA_DOB; **(I)** HLA_F.

Supplementary Figure S10 | The survival curves of the remaining 9 genes in the GSE140082 cohort. **(A)** BATF2; **(B)** CCR7; **(C)** CD3G; **(D)** CD40LG; **(E)** CLEC5A; **(F)** CXCL9; **(G)** ETV7; **(H)** HLA_DOB; **(I)** HLA_F.

Supplementary Figure S11 | The survival curves of the remaining 9 genes in the GSE32062 cohort. **(A)** BATF2; **(B)** CCR7; **(C)** CD3G; **(D)** CD40LG; **(E)** CLEC5A; **(F)** CXCL9; **(G)** ETV7; **(H)** HLA_DOB; **(I)** HLA_F.

Supplementary Figure S12 | The lollipop charts showed the correlation between the immune infiltrates and **(A)** CD38 and **(B)** CXCL13 expression in the TCGA-OV cohort.
